# Towards Targeted Alpha Therapy with Actinium-225: Chelators for Mild Condition Radiolabeling and Targeting PSMA—A Proof of Concept Study

**DOI:** 10.3390/cancers13081974

**Published:** 2021-04-20

**Authors:** Falco Reissig, David Bauer, Kristof Zarschler, Zbynek Novy, Katerina Bendova, Marie-Charlotte Ludik, Klaus Kopka, Hans-Jürgen Pietzsch, Milos Petrik, Constantin Mamat

**Affiliations:** 1 Helmholtz-Zentrum Dresden-Rossendorf, Institute of Radiopharmaceutical Cancer Research, Bautzner Landstraße 400, D-01328 Dresden, Germany; f.reissig@hzdr.de (F.R.); BauerD1@mskcc.org (D.B.); m.ludik@hzdr.de (M.-C.L.); k.kopka@hzdr.de (K.K.); h.j.pietzsch@hzdr.de (H.-J.P.); 2 Faculty of Chemistry and Food Chemistry, Technische Universität Dresden, D-01062 Dresden, Germany; 3 Institute of Molecular and Translational Medicine, Faculty of Medicine and Dentistry, Palacky University Olomouc, Hnevotinska 1333/5, 779 00 Olomouc, Czech Republic; zbynek.novy@upol.cz (Z.N.); katerina.bendova01@upol.cz (K.B.); milos.petrik@upol.cz (M.P.)

**Keywords:** actinium-225, PSMA, theranostics, targeted alpha therapy, click chemistry

## Abstract

**Simple Summary:**

In nuclear medicine, therapeutic methods are used increasingly, in which tumors are destroyed by ionizing radiation that cannot or can only be treated insufficiently by other methods like surgery or chemotherapy. Targeted alpha therapy (TAT) is a promising method with increasing importance that facilitates new treatment options for advanced and late-stage cancer diseases. The effectiveness of alpha-emitting radionuclides is characterized by a higher linear energy transfer and a higher biological efficacy, compared to therapeutic approaches with beta emitters. ^225^Ac is an alpha emitter with favorable nuclear properties for radiopharmaceutical applications. The aim of our research was to find a universal chelator that enables the attachment of sensitive bio(macro)molecules and allows ^225^Ac-radiolabeling under mild conditions. An aza-macrocycle-derived mcp chelator with functional groups for universal connection of biomolecules using convenient click chemistry was developed for the ^225^Ac-labeling. The resulting ^225^Ac-radioconjugates were analyzed in vitro and in vivo, showing a high receptor affinity on tumor cells and a high tumor accumulation in tumor-bearing mice.

**Abstract:**

Currently, targeted alpha therapy is one of the most investigated topics in radiopharmaceutical cancer management. Especially, the alpha emitter ^225^Ac has excellent nuclear properties and is gaining increasing popularity for the treatment of various tumor entities. We herein report on the synthesis of two universal ^225^Ac-chelators for mild condition radiolabeling and binding to conjugate molecules of pharmacological interest via the copper-mediated click chemistry. A convenient radiolabeling procedure was investigated as well as the complex stability proved for both chelators and two PSMA (prostate-specific membrane antigen)-targeting model radioconjugates. Studies regarding affinity and cell survival were performed on LNCaP cells followed by biodistribution studies, which were performed using LNCaP tumor-bearing mice. High efficiency radiolabeling for all conjugates was demonstrated. Cell binding studies revealed a fourfold lower cell affinity for the PSMA radioconjugate with one targeting motif compared to the radioconjugate owing two targeting motifs. Additionally, these differences were verified by in vitro cell survival evaluation and biodistribution studies, both showing a higher cell killing efficiency for the same dose, a higher tumor uptake (15%ID/g) and a rapid whole body clearance after 24 h. The synthesized chelators will overcome obstacles of lacking stability and worse labeling needs regarding ^225^Ac complexation using the DOTA (1,4,7,10-tetraazacyclododecane-1,4,7,10-tetrayl)tetraacetic acid) chelator. Moreover, the universal functionalization expands the coverage of these chelators in combination with any sensitive bio(macro)molecule, thus improving treatment of any addressable tumor target.

## 1. Introduction

Targeted alpha therapy (TAT) provides new treatment options for advanced and late-stage cancer diseases but also offers new possibilities for radiopharmaceutical cancer research. Compared to other therapies (e.g., external radiotherapy and radioligand therapy (RLT) based on beta emitters), TAT is putatively more efficient in eliciting cell death due to the exceedingly high ionizing density. The short range of alpha particles minimizes the unwanted irradiation for surrounding healthy tissue and reduces possible side effects [1]. Unfortunately, only a few alpha-emitting radionuclides are currently available, owing appropriate qualities for medical applications.

Besides the alpha-emitting radionuclide astatine-211, which is covalently bound to the targeting moiety [2,3,4,5], today’s focus lies on radiometals, particularly on bismuth and radium radionuclides, as well as on thorium-227 and actinium-225 [6,7]. Each radiometal has its advantages and obstacles. Bismuth-212 (t_1/2_ = 61 min) or the lead-212/bismuth-212 in vivo generator system and bismuth-213 (t_1/2_ = 46 min) showed promising results in clinical and preclinical studies [8,9]. Still, there is a lack of applications, most likely induced by the high amount of administered activities resulting in adverse reactions and a loss of flexibility due to the short half-lives. Radium-223 (t_1/2_ = 11 d) and radium-224 (t_1/2_ = 3.6 d) possess suitable physical properties by emitting multiple alpha particles in their decay chains with total decay energies of about 30 MeV resulting in a high linear energy transfer. Xofigo^®^ is the only clinically relevant application with EMA- and FDA-approval for radium-223 to date and is used in the form of [^223^Ra]RaCl_2_ to treat metastatic prostate cancer [10]. The major limitation of radium is the lack of an organic or inorganic framework to bind this radionuclide stably [11]. Despite several approaches to coordinate Ra^2+^, (e.g., using macrocyclic ligands [12,13,14,15], inorganic nanomatrices such as nanoparticles based on BaSO_4_ [16,17], barium ferrite [18], TiO_2_ and hydroxyapatite [19,20] or polyoxometalates [21]), an ideal carrier was not found, yet.

During the last decade, the radiometals actinium-225 (t_1/2_ = 10 d) and thorium-227 (t_1/2_ = 19 d) gained relevance as promising alpha emitters suitable for future targeting radiotherapeutics. Thorium-227-labeled antibody-Me-3,2-HOPO-conjugates are currently investigated in four clinical phase I trials [22,23]. Eleven clinical trials are displayed for actinium-225-DOTA-based conjugates. A trial of [^225^Ac]Ac-PSMA-617 in men with PSMA-positive prostate cancer is projected to start this year [24]. Actinium-225 can easily be complexed with the DOTA chelator, but requires high temperatures (80–95 °C) or microwave assistance [23]. However, first studies showed that the chelator macropa (mcp) might be even more suitable [25]. This diaza-18-crown-6-based chelator, substituted with two picolinate moieties, is known to form exceedingly stable actinium complexes, requiring mild conditions and ambient temperature for labeling [26]. Remarkably, complexation at ambient temperature allows direct radiolabeling of even heat-sensitive bio(macro)molecules such as antibodies and antibody fragments [27].

Our recent work focuses on new macropa-based ligands for the development of actinium-225-conjugates, combining both the extremely high complex stability and a clickable functionalization site for attaching biological targeting molecules. We aimed to synthesize a chelator, which can be functionalized for conjugations using click chemistry by the [3+2] Huisgen cycloaddition approach (CuAAC). For this purpose, we designed two propargyl-functionalized mcp-chelators based on the diaza-18-crown-6-skeleton. In a preclinical proof-of-concept-study, this chelator was conjugated to an azide-functionalized carboxylic acid as well as an azide-functionalized PSMA-binding motif. We compared the radiolabeling efficiency of our new chelators and their PSMA conjugates, performed cell-binding affinity and colony survival assays on PSMA-positive human prostate adenocarcinoma (LNCaP) cells. Finally, both new tracers were characterized regarding biodistribution in healthy and LNCaP tumor-bearing mice.

## 2. Results

### 2.1. Chelator Development

**Scheme 1:**
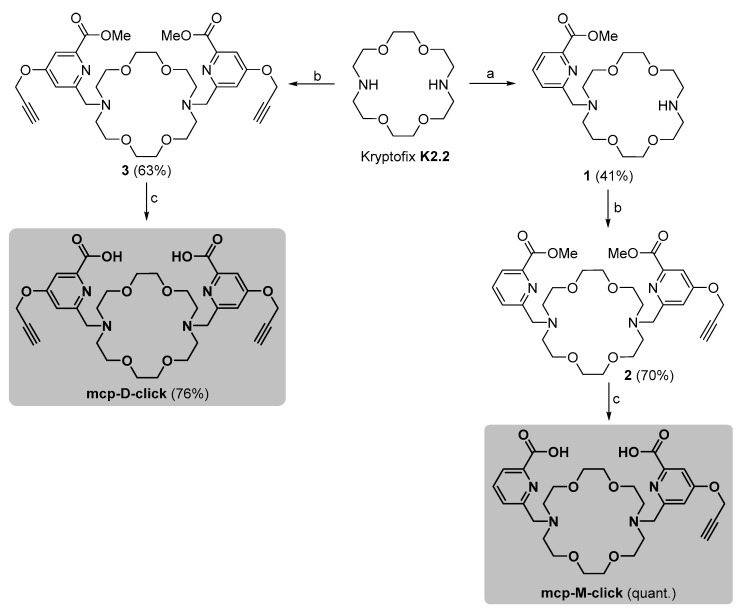
Synthesis for the macropa-derived, alkyne-functionalized chelators **mcp-M-click** and **mcp-D-click**. Reaction conditions: (**a**) Methyl 6-(chloromethyl)picolinate, *N*,*N*-diisopropylethylamine, acetonitrile, reflux, overnight; (**b**) methyl 6-(chloromethyl)-4-(prop-2-yn-1-yloxy)picolinate (**SI-III**, see Scheme S1), *N*,*N*-diisopropylethylamine, acetonitrile, reflux, overnight; (**c**) lithium hydroxide, water/methanol, 50 °C, 4 h.

Our new targeting conjugates for an improved radiolabeling with actinium-225 are based on a macropa-derived chelator and the well-characterized targeting biomolecule PSMA-617. We designed two chelators functionalized either with one or two alkyne moieties for an easy and quick conjugation with biomolecules of interest using the copper-catalyzed azide-alkyne-cycloaddition reaction [28]. For comparison, we synthesized the chelator macropa according to previously published procedures [29]. The synthesis of the novel mono- and di-alkyne-modified chelators **mcp-M-click** and **mcp-D-click** is presented in Scheme 1.

Both novel universal clickable chelators are based on 4,13-diaza-18-crown-6 (Kryptofix **K2.2**). For the monofunctionalization, **K2.2** was reacted with methyl 6-(chloromethyl)picolinate to give the diazacrown derivative **1**. To introduce the clickable alkyne-function, compound **1** was reacted with methyl 6-(chloromethyl)-4-(prop-2-yn-1-yloxy)picolinate (**SI-III**). The preparation of **SI-III** is expressed in the Supporting Information, Scheme S1 and Figures S1–S3). The resulting dimethyl ester **2** was finally saponified with lithium hydroxide to provide the monoalkyne chelator **mcp-M-click**. To obtain the bis-alkyne derivative **mcp-D-click**, **K2.2** was reacted with **SI-III** in excess to yield the respective symmetric diester **3** with two alkyne functions, which was finally saponified with lithium hydroxide.

**Scheme 2:**
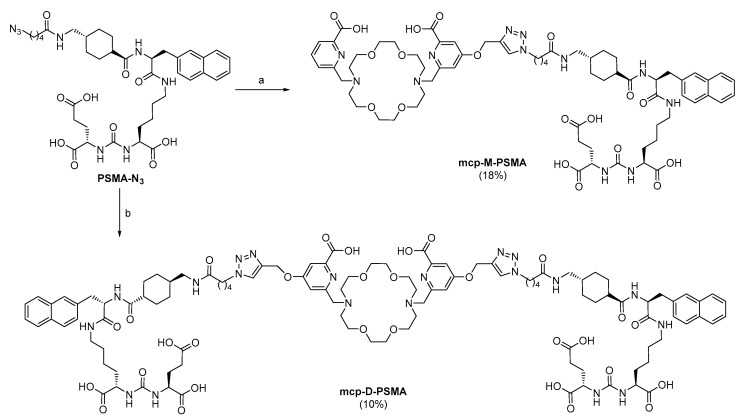
Synthesis of the monomeric and dimeric PSMA-targeting structure **mcp-M-PSMA** and **mcp-D-PSMA**. Reaction conditions: (**a**) **mcp-M-click**, [(CH_3_CN)_4_Cu]PF_6_, tris[(1-benzyl-1*H*-1,2,3-triazol-4-yl)methyl]amine, *N*,*N*-dimethylformamide, room temperature, overnight; (**b**) **mcp-D-click**, [(CH_3_CN)_4_Cu]PF_6_, tris[(1-benzyl-1*H*-1,2,3-triazol-4-yl)methyl]amine, *N*,*N*-dimethylformamide, room temperature, overnight.

Both PSMA conjugates **mcp-M-PSMA** and **mcp-D-PSMA** were finally synthesized under click chemistry conditions (CuAAC) by incubating the chelators **mcp-M-click** and **mcp-D-click** with the PSMA building block **PSMA-N_3_** (Scheme 2). The synthesis of the click building block **PSMA-N_3_**, which is based on the PSMA-617 binding motif, is described in the Supporting Information (Scheme S3, Figures S5 and S6). In addition, the model chelator **mcp-M-COOH** was prepared under the conditions mentioned before (see SI, Scheme S2 and Figure S4).

### 2.2. Radiolabeling and Complex Stability

To compare the efficiency of the synthesized chelators and their conjugates, 100 kBq of actinium-225 were incubated with each ligand at different concentrations (10^−5^ M; 10^−6^ M; 10^−7^ M). The radiochemical yield was determined after one hour of reaction time using normal phase and reversed-phase TLC. Whereas one TLC-system was designed to move free actinium-225 with the solvent front, the other system was developed only to move the actinium-225 labeled complexes. Concentration-dependent radiochemical yields for all ligands and 7-day-longterm yields, which were calculated using both TLC systems, are presented in Table 1.

All investigated ligands show excellent complex formation abilities with [^225^Ac]Ac^3+^, even at the low ligand concentration of 10^−7^ M. No activity-release was detected over seven days. Furthermore, the improved labeling yields obtained at the lowest concentrations after seven days showing that complexation might not be finished after one hour. Comparing **mcp-M-click** and **mcp-D-click** with the non-functionalized reference ligand macropa, the slight difference in the affinity might result from the steric hindrance caused by the propargyl moiety. A further enhancement even in yields at low concentrations and long-time stabilities was found using the model conjugate [^225^Ac]Ac-**mcp-M-COOH** containing an additional triazole moiety, presumably due to the additional nitrogen donor atom. [^225^Ac]Ac-**mcp-M-COOH** thereby predicts excellent labeling properties for the PSMA conjugates as well. Higher yields were obtained as well using the PSMA-functionalized conjugates **mcp-M-PSMA** and **mcp-D-PSMA**.

**Figure 1:**
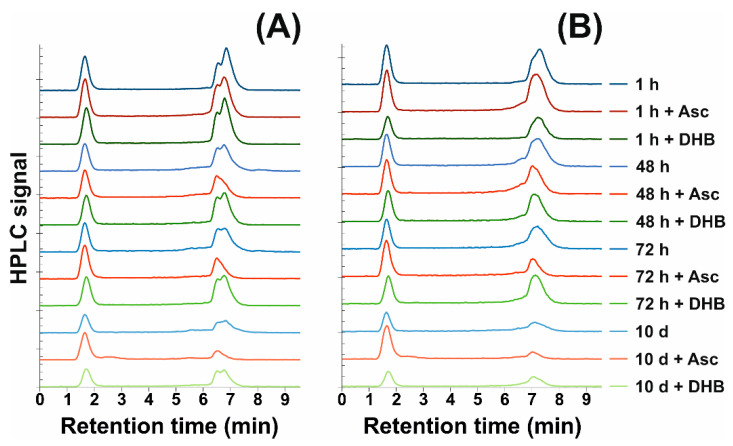
Radio-HPLC chromatograms for radiolabeled **mcp-M-PSMA** (**A**) and **mcp-D-PSMA** (**B**) in acetate buffer, pH 6 at room temperature in the absence and presence of stabilizers as a function of time. Samples were incubated with or without 0.1 M ascorbic acid (Asc) or 0.1 M 2,5-dihydroxy benzoic acid (DHB), respectively.

Unfortunately, a suitable reversed-phase iTLC system for the ligands **mcp-M-PSMA** and **mcp-D-PSMA** was not found so far to determine the radiochemical yields. The resolution of our standard system was not efficient, most likely due to the bigger size of these molecules compared to the basic chelators, different protonation steps and possible conformational structures. Additionally, logD_7.4_ values were determined to be −2.8 ± 0.1 for the complex [^225^Ac]Ac-**mcp-M-PSMA** and −2.5 ± 0.1 for the complex [^225^Ac]Ac-**mcp-D-PSMA**, respectively. Additionally, the stability of the ^225^Ac-labeled conjugates in acetate buffer was investigated by HPLC analyses over a period of ten days. Representative chromatograms of [^225^Ac]Ac-**mcp-M-PSMA** and [^225^Ac]Ac-**mcp-D-PSMA** with and without the addition of the antioxidant excipients ascorbic acid (Asc) or 2,5-dihydroxybenzoic acid (DHB), respectively, are shown in Figure 1.

The radio-HPLC-chromatograms in Figure 1 show two to three signals originating from radionuclides of the actinium-225 decay chain. Consequently, analyses of the respective HPLC fractions were performed using a high resolution gamma detector. The peak with a retention time of ~1.8 min was identified to be uncomplexed francium-221. The other two signals were identified as a mixture of [^213^Bi]Bi- and [^225^Ac]Ac-labeled PSMA conjugates.

Regarding the intensively discussed side-effects that are often caused by daughter nuclides, this could be an additional advantage when the chelators form stable complexes with both metal ions actinium-225 and bismuth-213. Nevertheless, recoiling daughter nuclides like bismuth-213 remains an aspect to be considered [30,31], since breaking of chemical bonds represents an inevitable result of alpha decay no matter which chelator is used.

Most importantly, radiolabeled **mcp-M-PSMA** as well as **mcp-D-PSMA** are stable in acetate buffer at room temperature for at least 48 h without antioxidants as demonstrated in Figure 1. However, the addition of 0.1 M DHB increases the stability of both PSMA derivatives by preventing radiolytic degradation to a certain extent. The presence of 0.1 M ascorbic acid did not stabilize the ^225^Ac-labeled conjugates and led to less complex stability and transchelation (verified by TLC). Although DHB appears to be more effective than ascorbic acid in retarding the dissociation by ionizing radiation, that might not apply for other ^225^Ac-based radiopharmaceuticals. Therefore, we conclude that the stability evaluation and stabilization needs to be assessed for each individual radioconjugate. In contrast to other previously published results on crown-based actinium-chelators [32], we do not conclude that actinium and alpha particle emitting radiopharmaceuticals in general, are only applicable in the first hours after production. To the best of our knowledge, future pharmaceuticals using our radioconjugate would not have to be produced in-house solely and possess adequate stability at an appropriate molar activity of 5 MBq/nmol (regarding the total ligand amount).

### 2.3. Characterization of the PSMA-Binding Properties

**Figure 2:**
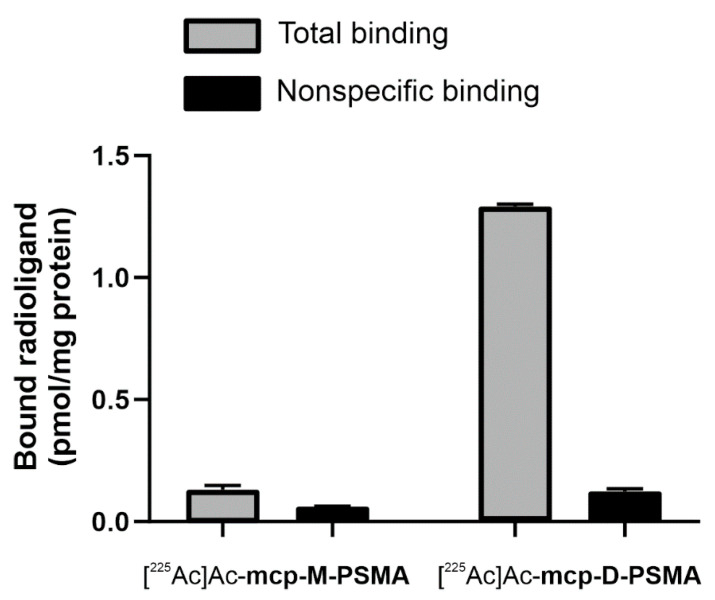
Binding specificity of [^225^Ac]Ac-**mcp-M-PSMA** and [^225^Ac]Ac-**mcp-D-PSMA** was determined by total binding (non-blocked) and nonspecific binding to PSMA-positive LNCaP cells, the latter by co-incubation with a 1000-fold molar excess of unlabeled PSMA-617 (*n* = 3).

Saturation binding studies using PSMA-positive LNCaP cells (see SI, Figures S14 and S15 for PSMA expression analysis) in combination with radiolabeled **mcp-M-PSMA** or **mcp-D-PSMA**, respectively, were performed to evaluate the binding specificity and affinity of the PSMA targeting variants. Incubation of [^225^Ac]Ac-**mcp-M-PSMA** or [^225^Ac]Ac-**mcp-D-PSMA** (30 nM) with LNCaP cells revealed a specific binding to PSMA, which was blocked significantly by the addition of an excess of PSMA-617 (Figure 2).

**Figure 3:**
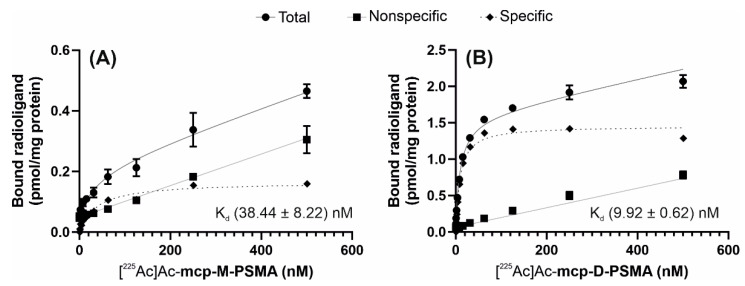
Saturation binding curves for ^225^Ac-labeled **mcp-M-PSMA** (**A**) and **mcp-D-PSMA** (**B**). Total binding was measured in the absence of and nonspecific binding in the presence of 500 µM unlabeled PSMA-617. Specific binding was calculated as the difference between total and nonspecific binding (*n* = 3 for each data point).

Specific binding of the dimeric compound [^225^Ac]Ac-**mcp-D-PSMA** was approximately ninefold higher than that of the monomeric counterpart [^225^Ac]Ac-**mcp-M-PSMA** (1.17 vs. 0.07 pmol/mg protein). After incubation of LNCaP cells with a serial dilution of [^225^Ac]Ac-**mcp-M-PSMA** or [^225^Ac]Ac-**mcp-D-PSMA**, respectively, the equilibrium dissociation constants K_d_ were calculated by the GraphPad Prism software using a “One site–total and nonspecific binding” analysis (Figure 3). A K_d_ of (38.44 ± 8.22) nM and a B_max_ of (0.163 ± 0.011) pmol/mg protein were determined for [^225^Ac]Ac-**mcp-M-PSMA**, whereas a K_d_ of (9.92 ± 0.62) nM and a B_max_ of (1.528 ± 0.023) pmol/mg protein were calculated for [^225^Ac]Ac-**mcp-M-PSMA**. The obtained data indicate an improved binding affinity of the dimeric derivative compared to the monomer. These results are in line with findings published by Schäfer et al. [33] and Frei et al. [34] revealing the outperforming cell binding and uptake properties of dimeric PSMA samples. The presence of two independent PSMA-binding motifs within the dimeric [^225^Ac]Ac-**mcp-D-PSMA** allows the compound to bind first with one motif to the respective antigen. The remaining (unoccupied) binding motif is now in close spatial proximity to other target sites on the cell surface and can bind to a second PSMA receptor. This divalent binding behavior stabilizes the [^225^Ac]Ac-**mcp-D-PSMA** interaction as transient unbinding of a single site does not necessarily result in the release of the captured compound unless the dissociation occurs at both sites [35]. Accordingly, the equilibrium dissociation constant of the monomeric [^225^Ac]Ac-**mcp-M-PSMA** is approximately three times higher than that of the dimeric compound, suggesting that monovalent binding is less stable than bivalent binding.

### 2.4. Cytotoxicity of ^225^Ac-Labeled PSMA Derivatives

**Figure 4:**
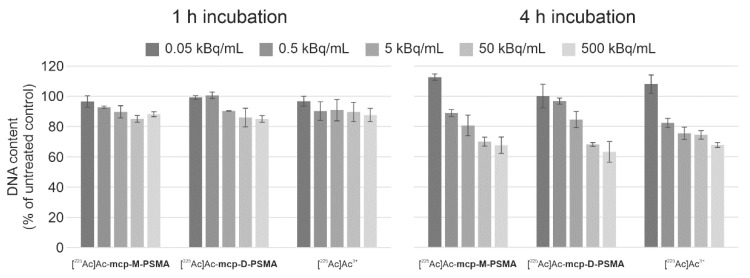
Antiproliferative activity of ^225^Ac-labeled PSMA derivatives compared to the non-targeted activity control. PSMA-expressing LNCaP cells were incubated for 1 or 4 h, respectively, with increasing activity concentrations of [^225^Ac]Ac-**mcp-M-PSMA**, [^225^Ac]Ac-**mcp-D-PSMA** or the non-targeted activity control [^225^Ac]Ac^3+^, and subsequently supplemented with fresh medium. The DNA fluorescence assay was performed 24 h after radioactivity removal.

To evaluate the cytotoxicity in vitro induced by [^225^Ac]Ac-**mcp-M-PSMA** or [^225^Ac]Ac-**mcp-D-PSMA**, LNCaP cells were exposed once for 1 or 4 h, respectively, and the cell number was determined 24 h after the treatment by DNA staining (Figure 4).

The relative number of LNCaP cells decreased with increasing radioactive doses and with extended incubation time. Already after 1 h of exposure, the proliferation rate was reduced by more than 10% at the second highest dose of 50 kBq/mL. The relative cell number is further decreased by approximately one third at the highest applied dose after 4 h of exposure to [^225^Ac]Ac-**mcp-M-PSMA** or [^225^Ac]Ac-**mcp-D-PSMA**. However, the incubation with [^225^Ac]Ac^3+^ as non-targeted activity control reduced the proliferation to a similar extent.

**Figure 5:**
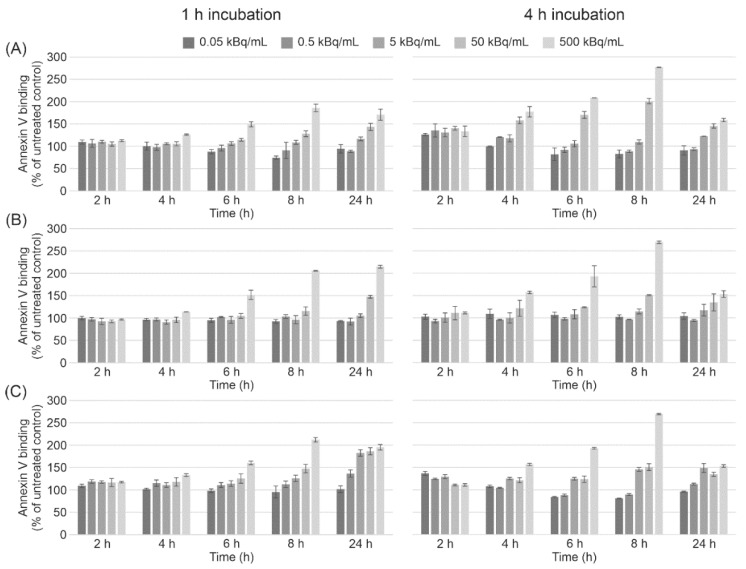
Kinetic profile of phosphatidylserine exposure on apoptotic LNCaP cells as a response to the treatment with ^225^Ac-labeled PSMA derivatives. The human prostate adenocarcinoma cells were incubated for 1 or 4 h, respectively, with increasing activity concentrations of [^225^Ac]Ac-**mcp-M-PSMA** (**A**), [^225^Ac]Ac-**mcp-D-PSMA** (**B**) or non-targeted [^225^Ac]Ac^3+^ (**C**), and subsequently supplemented with fresh medium. The real-time, bioluminescent annexin V assay was performed 2, 4, 6, 8 and 24 h after radioactivity removal.

To determine whether the antiproliferative activity of the ^225^Ac-labeled PSMA derivatives is associated with triggering programmed cell death pathways, the induction of apoptosis was analyzed by measuring the level of phosphatidylserine exposure on the outer leaflet of the cell membrane using an annexin V based assay (Figure 5). The incubation of human prostate adenocarcinoma cells with the ^225^Ac-labeled PSMA derivatives or with non-targeted [^225^Ac]Ac^3+^, respectively, resulted in increased staining for exposed phosphatidylserine beginning at 4 h, indicating the induction of apoptosis. The magnitude of annexin V binding grew steadily until 8 h owing to more phosphatidylserine exposure by the progression of the cellular apoptotic program. Noteworthy, no appreciable loss of membrane integrity due to secondary necrosis was detected during this period (data not shown). Phosphatidylserine exposure appears dose- and time-dependent, whereas no substantial difference was observed between the treatment with [^225^Ac]Ac-**mcp-M-PSMA,** [^225^Ac]Ac-**mcp-D-PSMA** or non-targeted [^225^Ac]Ac^3+^. This is in agreement with the data obtained by the proliferation assay and suggests that ^225^Ac-induced cell damage is independent of PSMA targeting. Obviously, [^225^Ac]Ac^3+^ added directly to the cell culture media appears to be as effective as the specifically binding ^225^Ac-labeled ligands. However, these observations originate from the experimental design. Under the static (flow-free) conditions of a two-dimensional cell culture in a simple petri dish, molecular targeting and cell binding via one or two PSMA-binding motifs plays no—or just a negligible—role for the efficacy of α particles coming from ^225^Ac and the direct radiotoxic effects of [^225^Ac]Ac-**mcp-M-PSMA,** [^225^Ac]Ac-**mcp-D-PSMA** and non-targeted [^225^Ac]Ac^3+^ are comparable.

### 2.5. Clonogenicity in Response to the Incubation with ^225^Ac-Labeled PSMA Derivatives

**Figure 6:**
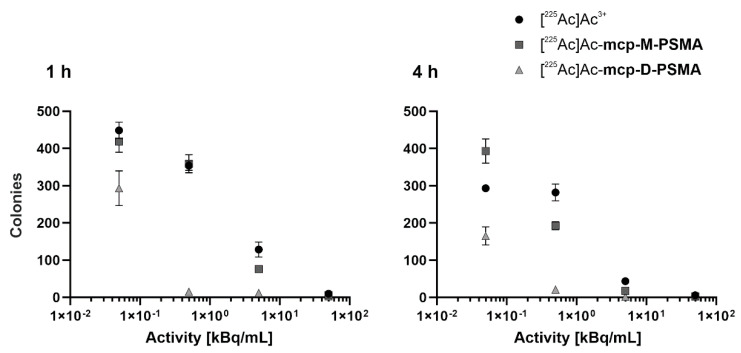
Colony-forming assay using LNCaP cells, indicating the ability of both ^225^Ac-labeled PSMA ligands to inhibit cell proliferation and colony formation in a time- and dose-dependent manner. The human prostate adenocarcinoma cells were incubated for 1 or 4 h, respectively, with increasing activity concentrations of [^225^Ac]Ac-**mcp-M-PSMA**, [^225^Ac]Ac-**mcp-D-PSMA** or [^225^Ac]Ac^3+^ as non-targeted activity control. After the indicated treatment time, cells were supplemented with fresh medium and cultures were incubated for 8 days to allow colony growth.

A colony formation assay, performed to assess the clonogenic survival of LNCaP cells after treatment with increasing activity (0.05 kBq–50 kBq/mL) of [^225^Ac]Ac-**mcp-M-PSMA** or [^225^Ac]Ac-**mcp-D-PSMA**, respectively, revealed a time- and dose-dependent decrease in colony-forming cells (see SI, Table S1). Furthermore, notable differences in the formation of colonies upon incubation with PSMA-targeted ([^225^Ac]Ac-**mcp-M-PSMA** and [^225^Ac]Ac-**mcp-D-PSMA**) and [^225^Ac]Ac^3+^ as non-targeted activity control were observed. The one-hour treatment with 5 kBq/mL resulted in 19.4% of the reference amount of colonies for the monomeric PSMA derivative and in 3.1% for the dimeric counterpart. In contrast, the clonogenic cell survival was higher (32.9%) after the treatment with the identical activity concentration of non-targeted [^225^Ac]Ac^3+^. Noteworthy, in terms of the cytotoxic capacity, the dimeric [^225^Ac]Ac-**mcp-D-PSMA** is superior to the monomeric [^225^Ac]Ac-**mcp-M-PSMA** at all time points and even at low activity levels (Figure 6).

**Figure 7:**
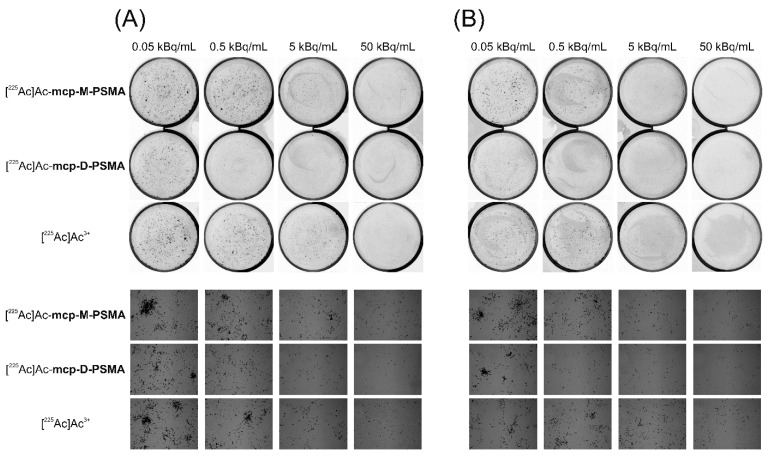
Representative overview photographs and microscopic images of crystal violet stained wells, after colony-forming assay using LNCaP cells, upon exposure to increasing activity concentrations (0.05 kBq–50 kBq/well) of [^225^Ac]Ac-**mcp-M-PSMA** and [^225^Ac]Ac-**mcp-D-PSMA**, compared to [^225^Ac]Ac^3+^ as non-targeted activity control for 1 h (**A**) or 4 h (**B**). After the indicated treatment time, cells were supplemented with fresh medium and cultures were incubated for eight days to allow colony growth (magnification: 40 ×).

Microscopic images of the crystal violet stained well plates underline the cytotoxic potency of both ^225^Ac-radioconjugates and highlight the increased cell-killing capacity of the dimeric complex compared to the monomeric ligand (Figure 7). Nearly no colonies were detected after a one-hour exposure of the human prostate adenocarcinoma cells with 50 kBq/mL [^225^Ac]Ac-**mcp-D-PSMA**. The treatment of LNCaP cells with 5 and 50 kBq/mL [^225^Ac]Ac-**mcp-D-PSMA**, respectively, for 4 h also resulted in almost 100% inhibition of colony formation. 

### 2.6. Small Animal Biodistribution Experiments

**Figure 8:**
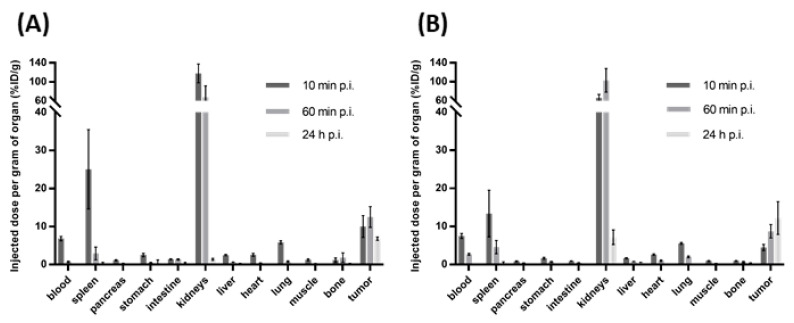
Biodistribution of [^225^Ac]Ac-**mcp-M-PSMA** (**A**) and [^225^Ac]Ac-**mcp-D-PSMA** (**B**) using LNCaP tumor-bearing SCID mice 10 min, 60 min and 24 h p.i. The data are presented as the mean of the percentage of injected dose per gram of organ ± standard deviation (*n* = 4).

The obtained biodistribution data showed similar in vivo behavior for both studied radiotracers [^225^Ac]Ac-**mcp-M-PSMA** and [^225^Ac]Ac-**mcp-D-PSMA** in LNCaP tumor-bearing mice (Figure 8; see Tables S2 and S3 in the SI for quantitative data). The biodistribution data using non-tumor-bearing SCID mice is presented in the SI (Figure S16, Tables S4 and S5).

The organ distribution profile indicates a PSMA-mediated uptake in tissues with documented PSMA expression including kidneys and spleen besides the pathologic radiotracer accumulation in the tumor [36,37,38,39]. The highest activity accumulation was found in kidneys at 10 min (117.81 ± 19.86%ID/g vs. 66.01 ± 7.20%ID/g) and 60 min (67.84 ± 23.92%ID/g vs. 103.03 ± 24.68%ID/g) p.i., rapidly decreasing in time (1.35 ± 0.26%ID/g vs. 7.17 ± 1.96%ID/g at 24 h p.i.) for both compounds. [^225^Ac]Ac-**mcp-D-PSMA** showed longer renal retention during the first 60 min p.i. compared to [^225^Ac]Ac-**mcp-M-PSMA**, what was expected due to the difference in molar masses, but also through the binding potential in the proximal tubuli. The renal clearance of both radiotracers from the blood was very fast and the majority of the activity was eliminated from the blood circulation within 60 min post injection. The other excised organs revealed negligible radioactivity uptake except the spleen at early time points. The radioactivity uptake in the spleen 10 min p.i. differs between the monomeric (25.05 ± 10.42 %ID/g) and the dimeric (13.42 ± 6.11 %ID/g) radioligand. For both ^225^Ac-labeled PSMA derivatives, the spleen uptake is substantially lower after 1 h p.i. with 2.90 ± 1.69 %ID/g and 4.57 ± 1.71 %ID/g, respectively. The observed difference in the spleen uptake 10 min p.i. originates from the individual blood clearance kinetics of both ligands as underlined by related differences in the kidney accumulation.

The tumor accumulation of [^225^Ac]Ac-**mcp-M-PSMA** was higher at 10 and 60 min p.i. compared to [^225^Ac]Ac-**mcp-D-PSMA**. The opposite results were observed at 24 h p.i., where [^225^Ac]Ac-**mcp-M-PSMA** showed a lower accumulation compared to [^225^Ac]Ac-**mcp-D-PSMA** (6.78 ± 0.45%ID/g versus 12.21 ± 4.31%ID/g). Tumor-to-background ratios were favorably high for both tested compounds and increased rapidly with time after administration. Tumor-to-blood ratios at 24 h p.i. were determined to be 188.42 and 158.11 for [^225^Ac]Ac-**mcp-M-PSMA** and [^225^Ac]Ac-**mcp-D-PSMA**, respectively, and tumor-to-muscle ratios at 24 h p.i. were calculated as 310.82 and 275.77. Overall, the biodistribution pattern of [^225^Ac]Ac-**mcp-M-PSMA** and [^225^Ac]Ac-**mcp-D-PSMA** is in accordance with previously published results for [^177^Lu]Lu-PSMA-617 or [^57^Co]Co-PSMA-617 [40,41] as well as with recent data for other small molecules targeting PSMA [27,39,42,43].

## 3. Materials and Methods

### 3.1. Chemistry

All chemicals were purchased from commercial suppliers and used without further purification. NMR spectra of all synthesized compounds were carried out on an Agilent DD2-400 MHz NMR or Agilent DD2-600 MHz NMR spectrometer with ProbeOne. All chemical shifts of ^1^H and ^13^C signals were reported in parts per million using TMS as internal standard at 25 °C and all spectra were calibrated using the respective solvent signal. Mass spectra were recorded on an Advion ExpressIon CMS or Xevo T-QS (Waters) using electrospray ionization. TLC analyses for reaction control were performed on Merck Silica Gel 60 F_254_ TLC plates and visualized using UV light. Analytical HPLC was performed on VWR Hitachi with an Agilent C18 column (Agilent Zorbax 300SB-C18, 100 mm × 4.6 mm) and acetonitrile/water (0.1% TFA each) as mobile phase. Chromatographic separations were performed using automated flash column chromatography on Isolera Four (Biotage) using silica gel cartridges (SNAP KP-Sil; 10 g or 25 g) and reversed phase HPLC system Knauer Azura with Zorbax 300SB-C18 semi-preparative column and acetonitrile/water (0.1% TFA each) as mobile phase. Starting materials such as anhydrous solvents, dimethyl 4-hydroxypyridine-2,6-dicarboxylate (TCI Europe) and 4,13-diaza-18-crown-6 (Merck) were purchased and used as received. Methyl 6-(chloromethyl)picolinate was prepared according to the literature [44].

#### 3.1.1. Methyl 6-((1,4,10,13-tetraoxa-7,16-diazacyclooctadecan-7-yl)methyl)picolinate **1**

4,13-Diaza-18-crown-6 (**K2.2**, 900 mg, 3.43 mmol) and *N*,*N*-diisopropylethylamine (399 mg, 3.09 mmol) were dissolved in anhydrous acetonitrile (30 mL) and brought to 80°C. Methyl 6-(chloromethyl)picolinate (450 mg, 2.42 mmol) dissolved in anhydrous acetonitrile (12 mL) was added dropwise by a syringe pump over a period of 5 h and the resulting mixture was refluxed overnight. After cooling to rt, the solids were removed by filtration and the solvent was removed. The crude product was purified via column chromatography (ethyl acetate/ethanol + 1% triethylamine) to obtain compound **1** as slightly yellow solid (580 mg, 41%). R*_f_* = 0.12 (ethyl acetate/ethanol/triethylamine = 10:15:0.5) and the bis-functionalized by-product dimethyl 6,6′-((1,4,10,13-tetraoxa-7,16-diazacyclooctadecane-7,16-diyl)bis(methylene))dipicolinate (183 mg; R*_f_* = 0.29); ^1^H NMR (600 MHz, CDCl_3_): δ = 2.75–2.85 (m, 4H, NCH_2_), 2.90–2.99 (m, 4H, NCH_2_), 3.40–3.60 (m, 12H, OCH_2_), 3.70–3.77 (m, 4H, OCH_2_), 3.86 (s, 2H, ArCH_2_), 3.96 (s, 3H, CH_3_), 7.87–8.00 (m, 3H, H_Ar_) ppm; ^13^C NMR (151 MHz, CDCl_3_): δ = 49.1 (NCH_2_), 52.9 (CH_3_), 56.2 (NCH_2_), 60.0 (ArCH_2_), 68.6 (OCH_2_), 68.9 (OCH_2_), 70.2 (OCH_2_), 70.3 (OCH_2_), 123.6 (CH_Ar_), 127.0 (CH_Ar_), 138.0 (CH_Ar_), 146.8 (C_Ar_), 161.4 (C_Ar_), 166.0 (C=O) ppm; MS (ESI+): *m/z* = 412 (100) [M+H]^+^, 434 (63) [M+Na]^+^. All original spectra can be found in the SI, Figure S7.

#### 3.1.2. Methyl 6-((16-((6-(methoxycarbonyl)pyridin-2-yl)methyl)-1,4,10,13-tetraoxa-7,16-diazacyclooctadecan-7-yl)methyl)-4-(prop-2-yn-1-yloxy)picolinate **2**

Compound **SI-III** (143 mg, 0.60 mmol), compound **1** (204 mg, 0.50 mmol) and *N*,*N*-diisopropylethylamine (107 μL, 0.60 mmol) were dissolved in anhydrous acetonitrile (10 mL) and the mixture was refluxed overnight. After cooling to rt and TLC control, the solvent was removed and the crude product was purified via column chromatography (ethyl acetate/ethanol + 1% triethylamine) to obtain compound **2** as colorless oil (214 mg, 70%). R*_f_* = 0.26 (ethyl acetate/ethanol = 2:3); ^1^H NMR (600 MHz, CDCl_3_): δ = 2.59 (br. s, 1H, ≡CH), 2.86 (br. s, 8H, NCH_2_), 3.52–3.68 (m, 12H, OCH_2_), 3.87–4.01 (m, 8H, 2 x CH_3_ + ArCH_2_), 4.78 (br. s, 2H, ArCH_2_), 7.47–7.60 (m, 2H, H_Ar_), 7.76 (t, ^3^*J* = 7.2 Hz, 1H, H_Ar_), 7.87 (br. s, 1H, H_Ar_), 7.94 (d, ^3^*J* = 7.2 Hz, 1H, H_Ar_) ppm; ^13^C NMR (151 MHz, CDCl_3_): δ = 53.0 (CH_3_), 53.1 (CH_3_), 54.5 (NCH_2_), 54.6 (NCH_2_), 56.0 (OCH_2_), 61.5 (br. s, OCH_2_), 69.9 (br. s, OCH_2_), 70.8 (OCH_2_), 70.9 (OCH_2_), 76.9 (≡C), 77.2 (≡CH), 110.9 (CH_Ar_), 112.0 (CH_Ar_), 123.6 (CH_Ar_), 126.4 (CH_Ar_), 137.5 (CH_Ar_), 147.2 (C_Ar_), 148.9 (C_Ar_), 161.5 (br. s, C_Ar_), 163.5 (br. s, C_Ar_), 165.3 (C_Ar_), 165.8 (C=O), 166.0 (C=O) ppm; MS (ESI+): *m/z* = 615 (100) [M+H]^+^, 636 (39) [M+Na]^+^. All original spectra can be found in the SI, Figure S8.

#### 3.1.3. Dimethyl 6,6′-((1,4,10,13-tetraoxa-7,16-diazacyclooctadecane-7,16-diyl)bis(methylene))bis(4-(prop-2-yn-1-yloxy)picolinate) **3**

4,13-Diaza-18-crown-6 (**K2.2**, 100 mg, 0.38 mmol), compound **SI-III** (228 mg, 0.95 mmol) and *N*,*N*-diisopropylethylamine (148 mg, 1.14 mmol) were dissolved in anhydrous acetonitrile (8 mL) and refluxed overnight. After cooling to rt, the solids were removed by filtration and the solvent was removed. The crude product was purified via column chromatography (ethyl acetate/ethanol + 1% triethylamine) to obtain compound **3** as slightly yellow solid (160 mg, 63%). R*_f_* = 0.35 (ethyl acetate/ethanol/triethylamine = 10:15:0.5); ^1^H NMR (600 MHz, CDCl_3_): δ = 2.59 (br. s, 2H, ≡CH), 2.85 (br. s, 8H, NCH_2_), 3.52–3.68 (m, 12H, OCH_2_), 3.84–4.02 (m, 10H, 2 x CH_3_ + NCH_2_), 4.78 (s, 4H, ArCH_2_), 7.46–7.61 (m, 4H, H_Ar_) ppm; ^13^C NMR (151 MHz, CDCl_3_): δ = 53.1 (CH_3_), 54.5 (NCH_2_), 54.6 (NCH_2_), 56.0 (OCH_2_), 61.5 (br. s, OCH_2_), 69.9 (br. s, OCH_2_), 70.9 (OCH_2_), 76.9 (≡C), 77.2 (≡CH), 110.9 (CH_Ar_), 112.1 (CH_Ar_), 148.9 (C_Ar_), 163.6 (br. s, C_Ar_), 165.3 (C_Ar_), 165.8 (C=O) ppm; MS (ESI+): *m/z* = 669 (100) [M+H]^+^, 691 (30) [M+Na]^+^. All original spectra can be found in the SI, Figure S9.

#### 3.1.4. 6-((16-((6-Carboxypyridin-2-yl)methyl)-1,4,10,13-tetraoxa-7,16-diazacyclooctadecan-7-yl)methyl)-4-(prop-2-yn-1-yloxy)picolinic acid **mcp-M-click**

Compound **2** (40 mg, 0.07 mmol) was dissolved in a 1/1 mixture of methanol and water (5 mL) and LiOH (10 mg, 0.42 mmol) was added. The resulting mixture was stirred at 50 °C for 4 h. Afterwards, the solvent was removed and purification was done via semi-preparative HPLC (eluent: 15% → 35% ACN + 0.1% TFA in 35 min, flow rate = 6 mL/min, t_R_ = 10 min; Agilent Zorbax 300SB-C18, 9.4 mm × 250 mm) to yield **mcp-M-click** as colorless solid (30 mg, 76%); MS (ESI+): *m/z* = 294 (100) [(M+2H)/2]^+^, 587 (47) [M+H]^+^, 609 (17) [M+Na]^+^. All original spectra can be found in the SI, Figure S10.

#### 3.1.5. 6,6′-((1,4,10,13-Tetraoxa-7,16-diazacyclooctadecane-7,16-diyl)bis(methylene))bis(4-(prop-2-yn-1-yloxy)picolinic acid) **mcp-D-click**

Compound **3** (100 mg, 0.15 mmol) was dissolved in a 1/1 mixture of methanol and water (5 mL) and LiOH (19 mg, 0.9 mmol) was added. The resulting mixture was stirred at 50 °C for 4 h. Afterwards, the solvent was removed and purification was done via semi-preparative HPLC (eluent: 15% → 35% ACN + 0.1% TFA in 35 min, flow rate = 6 mL/min, t_R_ = 16 min, Agilent Zorbax 300SB-C18, 9.4 mm × 250 mm) to yield **mcp-D-click** as colorless oil (90 mg, quant.); MS (ESI+): *m/z* = 321 (100) [(M+2H)/2]^+^, 641 (50) [M+H]^+^, 663 (10) [M+Na]^+^. All original spectra can be found in the SI, Figure S11.

#### 3.1.6. (3*RS*,10*RS*,14*S*)-1-((1*s*,4*S*)-4-((5-(4-(((2-Carboxy-6-((16-((6-carboxypyridin-2-yl)methyl)-1,4,10,13-tetraoxa-7,16-diazacyclooctadecan-7-yl)methyl)pyridin-4-yl)oxy)methyl)-1*H*-1,2,3-triazol-1-yl)pentanamido)methyl)cyclohexyl)-3-(naphthalen-2-ylmethyl)-1,4,12-trioxo-2,5,11,13-tetraazahexadecane-10,14,16-tricarboxylic acid **mcp-M-PSMA**

The chelator **mcp-M-click** (20 mg, 34 µmol), **PSMA-N_3_** (20 mg, 25 µmol), tetrakis(acetonitrile)copper(I) hexafluorophosphate (1.9 mg, 0.5 µmol) and tris[(1-benzyl-1*H*-1,2,3-triazol-4-yl)methyl]amine (2.7 mg, 0.5 µmol) were dissolved in anhydrous *N*,*N*-dimethylformamide (2 mL) and stirred at room temperature overnight. Afterwards, the solvent was removed and the crude product was purified by semi-preparative HPLC (eluent: 15% → 40% ACN + 0.1% TFA in 35 min, flow rate = 6 mL/min, t_R_ = 21 min, Agilent Zorbax 300SB-C18, 9.4 mm × 250 mm) to yield **mcp-M-PSMA** as colorless product (8.5 mg, 18%); MS (ESI+): *m/z* = 456 (24) [(M+3H)/3]^+^, 684 (100) [(M+2H)/2]^+^, 1367 (18) [M+H]^+^ (see SI, Figure S12).

#### 3.1.7. Bis-Functionalized PSMA Conjugate **mcp-D-PSMA**

The chelator **mcp-D-click** (20 mg, 31 µmol), **PSMA-N_3_** (46 mg, 58 µmol), tetrakis(acetonitrile)copper(I) hexafluorophosphate (2.2 mg, 0.6 µmol) and tris[(1-benzyl-1*H*-1,2,3-triazol-4-yl)methyl]amine (3.2 mg, 0.6 µmol) were dissolved in anhydrous *N*,*N*-dimethylformamide (2 mL) and stirred at room temperature overnight. Afterwards, the solvent was removed and the crude product was purified by semi-preparative HPLC (eluent: 25% → 80% ACN + 0.1% TFA in 35 min, flow rate = 6 mL/min, t_R_ = 10 min, Agilent Zorbax 300SB-C18, 9.4 mm × 250 mm) to yield **mcp-D-PSMA** as colorless product (6.5 mg, 10%); MS (ESI+): *m/z* = 735 (100) [(M+3H)/3]^+^, 1102 (11) [(M+2H)/2]^+^ (see SI, Figure S13).

### 3.2. Radiolabeling

Actinium-225 was purchased as a 0.1 M HCl solution from ITM Munich and used without purification. Stem solutions of all ligands were prepared in concentrations of 10^−2^ M and diluted to 10^−6^ M using 0.2 M ammonium acetate buffer at pH 6. For concentration-dependent labeling experiments, 10 µL of each ligand stem solution was incubated with 100 kBq of actinium-225 (1–5 µL) and the total volume was adjusted to 100 µL with 0.2 M ammonium acetate buffer at pH 6. For stability analyses, 50 µL of the buffer volume were replaced by 50 µL of 0.2 M ascorbic acid or 50 µL of 2,5-dihydroxy benzoic acid, respectively. Labeling for cell-binding studies was performed using 1.5 MBq of actinium-225 and 4 nmol of the respective PSMA ligand in a total volume of 400 µL adjusted with 0.2 M ammonium acetate buffer at pH 6. Radiolabeling for cell survival studies and all in vivo experiments were performed using 1.5 MBq of actinium-225 and 3.3 nmol of the respective ligand.

All samples were incubated for one hour at room temperature and analyzed afterward. TLC analyses were carried out in two different systems, normal phase on silica plates in 50 mM EDTA solution (pH 7) and reversed-phase on Alugram RP-CN in acetonitrile/water (7/3). TLC plates were imaged using a phosphoimager Amersham Typhoon 5 Scanner (Cytiva Europe GmbH, Freiburg, Germany) after a decay time of at least 7 h. Analytical HPLC was performed using a Jasco HPLC system with RITA analyzer (Elysia-raytest GmbH, Straubenhardt, Germany) for gamma detection on a Phenomenex Phenyl-Hexyl column in water/acetonitrile (+ 0.1% TFA). Radioactivity was measured in Isomed 2100 dose calibrator. Low radioactivity count rates were determined in Isomed 2160 sodium iodide detector. More precise measurements and nuclide identification were carried out on an activity- and efficiency-calibrated HPGe detector CryoPulse 5 (Canberra).

### 3.3. Cell Culture

The human prostate adenocarcinoma cell line LNCaP was obtained from the CLS Cell Lines Service (#300265, CLS Cell Lines Service GmbH, Eppelheim, Germany) and cultured in RPMI-1640 medium (Thermo Fisher Scientific, Waltham, MA, USA) supplemented with 10% fetal calf serum (FCS, Merck KGaA, Darmstadt, Germany). Cells were grown in a humidified atmosphere of 95% air/5% CO_2_ at 37 °C. For harvesting, counting and sub-cultivation, culture media was removed and cells were washed three times with PBS (Merck KGaA, Darmstadt, Germany). After the addition of 1% trypsin-EDTA (Merck KGaA, Darmstadt, Germany), cells were incubated for 5 min at 37 °C for detachment from the bottom of the culture flask. Then, culture media was added and cell suspension was transferred into conical centrifuge tubes and spun down for 5 min at 200 × g and room temperature. The cell pellet was resuspended in culture media and the cell number, as well as viability, was determined using a CASY cell counter (Roche Diagnostics GmbH, Penzberg, Germany) according to the manufacturer’s protocol.

### 3.4. Protein Determination

Protein concentration was determined with the DC Protein Assay (Bio-Rad Laboratories GmbH, Feldkirchen, Germany) according to the manufacturer’s microplate assay protocol using bovine serum albumin as a protein standard.

### 3.5. Gel Electrophoresis and Western Blot Analysis

Denaturing sodium dodecyl sulfate-polyacrylamide gel electrophoresis (SDS-PAGE) was performed according to a standard protocol [45]. For each gel, the PageRuler Plus Prestained Protein Ladder (Thermo Fisher Scientific, Waltham, MA, USA) was used as size standard. 

LNCaP cell lysates were prepared by addition of 1 mL of radioimmunoprecipitation assay (RIPA) cell lysis buffer (G-Biosciences, St. Louis, MO, USA) with EDTA-free protease and phosphatase inhibitor cocktail (Thermo Fisher Scientific, Waltham, MA, USA) and 25 U endonuclease (Thermo Fisher Scientific, Waltham, MA, USA). After resuspension and incubation on ice for at least 15 min, cell lysates were centrifuged at 14,000× *g* and 4 °C for 20 min. Equal protein amounts (10 μg) of the supernatants were separated on a 12% SDS polyacrylamide gel and transferred onto a polyvinylidene difluoride (PVDF) membrane (Merck KGaA, Darmstadt, Germany). Membranes were probed with primary anti-PSMA (D4S1F) and anti-β-actin (13E5) monoclonal rabbit antibodies (Cell Signaling Technology Europe B.V., Frankfurt am Main, Germany), respectively. As secondary antibodies, goat anti-rabbit horseradish peroxidase (HRP) conjugates (Merck KGaA, Darmstadt, Germany) were used. For detecting HRP on immunoblots, the WesternSure PREMIUM Chemiluminescent Substrate (LI-COR Biosciences-GmbH, Bad Homburg, Germany) in combination with the C-DiGit Blot Scanner and the Image Studio software (LI-COR Biosciences-GmbH, Bad Homburg, Germany) was used.

### 3.6. Saturation Binding Studies

Saturation binding studies were performed according to a published procedure [46]. LNCaP cells were plated in 48 well culture microplates (Greiner Bio-One GmbH, Frickenhausen, Germany) at a density of 3 × 10^4^ cells/250 µL/well and incubated for 48 h. Before addition of [^225^Ac]Ac-**mcp-M-PSMA** and [^225^Ac]Ac-**mcp-D-PSMA**, cells were pre-incubated for 30 min at 4 °C. After adding a serial dilution (15 pM–500 nM) of the ^225^Ac-labeled ligands, cells were incubated for 90 min at 4 °C. The nonspecific binding was assessed in parallel by blocking the cells with a 1000-fold excess of unlabeled PSMA-617. After the incubation time, the cells were washed three times with ice-cold PBS. Finally, cells were lysed by adding 1% SDS/0.1 M NaOH and shaking for 30 min at room temperature. To quantify the radioactivity in the cell extracts, an automatic gamma counter (Hidex Deutschland Vertrieb GmbH, Mainz, Germany) was used.

### 3.7. Cell Viability, Apoptosis and Cell Proliferation Assays

The treatment response on cellular proliferation was assessed using the CyQUANT direct cell proliferation assay (Thermo Fisher Scientific, Waltham, MA, USA). For this purpose, LNCaP cells were plated in black 96 well cell culture microplates (Greiner Bio-One GmbH, Frickenhausen, Germany) at a density of 2 × 10^4^ cells/0.05 mL/well. After 24 h, five different activity concentrations (0.05, 0.5, 5, 50 and 50 kBq/mL) of the ^225^Ac-labeled ligands [^225^Ac]Ac-**mcp-M-PSMA** and [^225^Ac]Ac-**mcp-D-PSMA** or the non-targeted activity control [^225^Ac]Ac^3+^ were diluted and added in triplicate so that the final volume was 0.1 mL/well. After 1 or 4 h of incubation, the cell culture media was removed and fresh media was added to each well. Cell numbers were subsequently determined 24 h later as per the manufacturer’s protocol. The fluorescence intensity was measured with the Varioskan Flash microplate reader (Thermo Fisher Scientific, Waltham, MA, USA). 

The exposure of phosphatidylserine on the extracellular side of cell membranes during the apoptotic process was measured using the RealTime-Glo™ Annexin V Apoptosis and Necrosis assay (Promega GmbH, Walldorf, Germany). To this end, LNCaP cells were plated in white 96 well cell culture microplates (Greiner Bio-One GmbH, Frickenhausen, Germany) at a density of 2 × 10^4^ cells/0.05 mL/well. After 24 h, five different activity concentrations (0.05, 0.5, 5, 50 and 50 kBq/mL) of the ^225^Ac-labeled ligands or the non-targeted activity control were diluted and added in triplicate so that the final volume was 0.1 mL/well. After 1 or 4 h of incubation, the cell culture media was removed and the assay reagent diluted in fresh media was added to each well. The fluorescence intensity and luminescence signals were measured for up to 24 h using the Varioskan Flash microplate reader (Thermo Fisher Scientific, Waltham, MA, USA).

### 3.8. Colony Formation Assay

LNCaP cells were plated in 6-well cell culture microplates (Greiner Bio-One GmbH, Frickenhausen, Germany) at a density of 6 × 10^3^ cells/2 mL/well. After 24 h, four different activity concentrations (0.05, 0.5, 5 and 50 kBq/mL) of the ^225^Ac-labeled ligands [^225^Ac]Ac-**mcp-M-PSMA** and [^225^Ac]Ac-**mcp-D-PSMA** were added in triplicate. After 1 or 4 h of incubation, the cell culture media was removed and fresh media was added to each well. Plates were then incubated at 37 °C for 8 days and re-fed every 3 to 4 days. Finally, the cell media was removed and colonies were stained with 1 mL of 0.5% crystal violet in 50% methanol for 30 min, after which the plates were rinsed three times with deionized water and subsequently air-dried. The plates were scanned with an Amersham Typhoon 5 Scanner (Cytiva Europe GmbH, Freiburg, Germany) and the colonies were counted using the Image-Quant TL software (Version 8.1, Cytiva Europe GmbH, Freiburg, Germany).

### 3.9. Animal Studies

The biodistribution studies with [^225^Ac]Ac-**mcp-M-PSMA** and [^225^Ac]Ac-**mcp-D-PSMA** were performed in LNCaP tumor-bearing mice. The SCID male mice (ENVIGO, Indianapolis, IN, USA) 6–8 weeks old were subcutaneously injected into the right flank with 10 × 10^6^ LNCaP cells (ATCC, Manassas, VA, USA) mixed with Matrigel Matrix (Corning, Corning, NY, USA) at a 1:1 ratio. The tumor growth was continuously monitored by caliper-based measurements. When the tumor volume reached 200–300 mm^3^ (i.e., 5–6 weeks after the inoculation of cells), the mice were used for biodistribution studies. The experimental animals were housed in a specific-pathogen-free animal facility. All experiments with animals were performed in accordance with appropriate legal norms (Czech Law No. 246/1992) and with the approval of the Ministry of Education, Youth and Sports (MSMT-35035/2019-3) and approval of the Ethical Committee of Faculty of Medicine and Dentistry, Palacky University in Olomouc. The number of animals was reduced as much as possible (*n* = 4 per group and time point) for all in vivo experiments.

For biodistribution studies, the radiotracers were diluted with saline. The prepared radiotracers were applied retro-orbitally (r.o.) to the experimental animals [47] at a dose of 50 kBq per mouse corresponding to 10 pmol of the peptide. The tracer injection was carried out under 2% isoflurane anesthesia (FORANE, Abbott Laboratories, Abbott Park, IL, USA) to minimize animal suffering and to prevent animal motion. The mice were sacrificed by cervical dislocation 10 min, 60 min and 24 h post-injection and organs of interest (blood, spleen, pancreas, stomach, intestine, kidneys, liver, heart, lungs, muscle, bone and tumor) were collected. The samples were weighed and the activity was measured on an automatic gamma counter. The radiotracer accumulation was expressed as a percentage of injected dose per gram (%ID/g) of the corresponding organ.

## 4. Conclusions

Two chelators for mild condition radiolabeling with ^225^Ac were developed, which enable a future use not only for the application of ^225^Ac-radiolabeled peptides, but also ^225^Ac-radiolabeled antibodies in targeted alpha therapy. In contrast to DOTA, both chelators revealed excellent labeling yields at low chelator concentrations after short labeling times at room temperature and appropriate long-term stabilities, as well. Additionally, the therapeutic efficiency in vitro and the applicability of two PSMA-targeting model radioconjugates on LNCaP tumor-bearing mice were shown in a proof of concept study on these triazole-containing click conjugates. The presented results provide bright prospects for various future applications for ^225^Ac in targeted alpha therapy, such as site-specific modification of bio(macro)molecules of the treatment of various types of cancer and a general improvement of DOTA-containing conjugates as well.

## Figures and Tables

**Table 1:** ^225^Ac-labeling of selected ligands in a serial dilution. Radiochemical yields (RCY) determined after 1 h of reaction time at room temperature and after seven days (NP: normal phase; RP: reversed-phase).

RCY (%)	[^225^Ac]Ac-macropa			[^225^Ac]Ac-mcp-M-click			[^225^Ac]Ac-mcp-D-click			[^225^Ac]Ac-mcp-M-COOH			[^225^Ac]Ac-mcp-M-PSMA			[^225^Ac]Ac-mcp-D-PSMA		
c [M]	10^−5^	10^−6^	10^−7^	10^−5^	10^−6^	10^−7^	10^−5^	10^−6^	10^−7^	10^−5^	10^−6^	10^−7^	10^−5^	10^−6^	10^−7^	10^−5^	10^−6^	10^−7^
NP (1 h)	99	99	56	99	99	20	99	99	52	99	99	98	99	99	89	99	99	93
NP (7 d)	99	99	99	99	99	58	99	99	50	99	99	99	99	99	99	99	99	95
RP (1 h)	99	90	37	96	97	23	99	99	41	99	98	98	/	/	/	/	/	/
RP (7 d)	99	99	99	99	99	55	99	94	40	99	99	96	/	/	/	/	/	/

## Data Availability

The data presented in this study are available in the Supplementary Materials.

## Supplementary Materials

The following are available online at https://www.mdpi.com/article/10.3390/cancers13081974/s1, Scheme S1: Synthesis overview for the preparation of alkyne-containing building block SI-III. Scheme S2: Synthesis of the model chelator **mcp-M-COOH** under click chemistry conditions. Scheme S3: Two-step synthesis approach for the click derivative **PSMA-N_3_**. Figure S1: ^1^H NMR and ^13^C NMR spectra of compound **SI-I**. Figure S2: ^1^H NMR and ^13^C NMR spectra of compound **SI-II**. Figure S3: ^1^H NMR and ^13^C NMR spectra of compound **SI-III**. **Figure S4**: ^1^H NMR spectrum of compound **mcp-M-COOH. Figure S5**: MS (ESI+) spectrum of compound **^t^Bu-PSMA-N_3_**. Figure S6: MS (ESI+) spectrum of compound **PSMA-N_3_**. Figure S7: ^1^H NMR, ^13^C NMR, COSY, HSQC, and MS (ESI+) spectra of compound **1**. Figure S8: ^1^H NMR, ^13^C NMR, COSY, HSQC, and MS (ESI+) spectra of compound **2**. Figure S9: ^1^H NMR, ^13^C NMR, COSY, HSQC, and MS (ESI+) spectra of compound **3**. Figure S10. ^1^H NMR, ^13^C NMR, and MS (ESI+) spectra of compound **mcp-M-click. Figure S11**: ^1^H NMR, ^13^C NMR, and MS (ESI+) spectra of compound **mcp-D-click**. Figure S12: Mass spectrum (ESI+) and expanded region of ligand **mcp-M-PSMA**. Figure S13: Mass spectrum (ESI+) and expanded region of ligand **mcp-D-PSMA. Figure S14**: Analysis of the prostate-specific membrane antigen (PSMA) expression in human prostate adenocarcinoma (LNCaP) cells. Figure S15: Analysis of the prostate-specific membrane antigen (PSMA) expression in HEK293, A431, LNCaP, PC3-PSCA, and PC3 cells. **Figure S16**: Biodistribution of [^225^Ac]Ac-**mcp-M-PSMA** (**A**) and [^225^Ac]Ac-**mcp-D-PSMA** (**B**) using healthy SCID mice 10 min. 60 min. and 24 h p.i. Table S1: Percentage amount of colonies referred to the non-radioactive control. Table S2: Quantitative evaluation of [^225^Ac]Ac-**mcp-M-PSMA** in LNCaP tumor-bearing SCID mice. The values are given as the mean of the percentage of injected dose per gram of organ ± standard deviation (*n* = 4). Table S3: Quantitative evaluation of [^225^Ac]Ac-**mcp-D-PSMA** in LNCaP tumor-bearing SCID mice. The values are given as the mean of the percentage of injected dose per gram of organ ± standard deviation (*n* = 4). Table S4: Quantitative evaluation of [^225^Ac]Ac-**mcp-M-PSMA** in healthy mice. Table S5: Quantitative evaluation of [^225^Ac]Ac-**mcp-D-PSMA** in healthy mice.
