# Supplementary material for: Towards Targeted Alpha Therapy with Actinium-225: Chelators for Mild Condition Radiolabeling and Targeting PSMA—A Proof of Concept Study
Source: Cancers (Basel). 2021 Apr 20;13(8):1974. doi: 10.3390/cancers13081974 (PMC8073976; doi:10.3390/cancers13081974)
Supplement: Supplementary file 1 [file cancers-13-01974-s001.zip › cancers-1152175-SI.pdf]

Supplementary Material: Towards Targeted Alpha Therapy with Actinium-225: Chelators for Mild Condition Radiolabeling and Targeting PSMA—A Proof of Concept Study

Falco Reissig, David Bauer, Kristof Zarschler, Zbynek Novy, Katerina Bendova, Marie-Charlotte Ludik, Klaus Kopka, Hans-Jürgen Pietzsch, Milos Petrik and Constantin Mamat

1. Synthesis of the Alkyne-Containing Building Block SI-III Including NMR Spectra

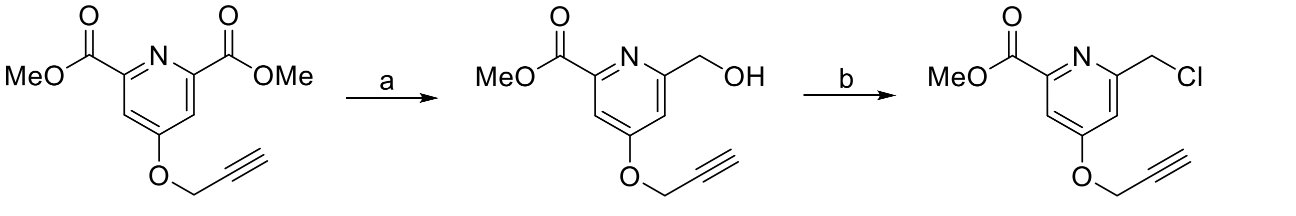

Scheme S1. Synthesis overview for the preparation of alkyne-containing building block **SI-III**.

1.1. Dimethyl 4-(prop-2-yn-1-yloxy)pyridine-2,6-dicarboxylate ***SI-I***

Dimethyl 4-hydroxypyridine-2,6-dicarboxylate (5.0 g, 20 mmol) was dissolved in anhydrous acetonitrile (50 mL), 3-bromo-1-propyne (2.2 g, 80% solution in THF) and K_2CO_3 (27.6 g, 200 mmol) were added and the reaction mixture was refluxed overnight. After cooling, the solids were filtered, the solvent was removed and the crude product was purified via column chromatography (chloroform → chloroform:ethyl acetate 10:1) to obtain compound **SI-I** as colorless solid (2.08 g, 96%). R_f = 0.34 (chloroform:ethyl acetate 10:1). The NMR and MS spectra were in accordance to the previously published (***J. Mater. Chem. B***, 2015, **3**, 1470-1473).

1.2. Methyl 6-(hydroxymethyl)-4-(prop-2-yn-1-yloxy)picolinate ***SI-II***

Compound **SI-I** (2.08 g, 8.35 mmol) was dissolved in anhydrous methanol (60 mL), $NaBH_4$ (346 mg, 9.15 mmol) was added and the mixture was stirred at 62°C for 5 h (TLC control). After cooling to rt, the solvent was removed, saturated $NaHSO_4$ solution (50 mL) and chloroform (50 mL) were added. The aqueous phase was extracted with chloroform (2 x 50 mL) and the combined organic phases were dried over $NaSO_4$. After removal of the solvent, the crude product was purified via column chromatography (chloroform → chloroform:ethyl acetate 5:1) to obtain compound **SI-II** as colorless solid (380 mg, 21%). R_f = 0.51 (chloroform:ethanol 10:1); ^{1}H NMR (400 MHz, $CDCl_3$): δ = 2.59 (t, ^4J = 2.4 Hz, 1H, ≡CH), 3.97 (s, 3H, CH_3), 4.79–4.82 (m, 4H, $ArCH_2$ + OCH_2), 7.13 (d, ^4J = 2.4 Hz, 1H, H_{Ar}), 7.62 (d, ^4J = 2.4 Hz, 1H, H_{Ar}) ppm; ^{13}C NMR (101 MHz, $CDCl_3$): δ = 53.1 (CH_3), 56.2 (OCH_2), 64.8 ($ArCH_2$), 76.8 (≡C), 77.2 (≡CH), 110.1 (CH_{Ar}), 111.4 (CH_{Ar}), 148.8 (C_{Ar}), 162.5 (C_{Ar}), 165.4 (C_{Ar}), 165.5 (C=O) ppm.

1.3. Methyl 6-(chloromethyl)-4-(prop-2-yn-1-yloxy)picolinate ***SI-III***

Compound **SI-II** (380 mg, 1.72 mmol) was cooled to 0°C and thionyl chloride (1 mL) was added slowly at this temperature. The mixture was stirred at 0°C for 1 h and at rt for 2 h. Afterwards, the mixture was added to a stirred solution of saturated $NaHCO_3$ solution (15 mL) at 0°C under vigorous stirring. Next, the solution was extracted with chloroform (3 x 15 mL) and the combined organic phases were dried over $NaSO_4$. After removal of

the solvent, compound **SI-III** was obtained as colorless solid (400 mg, 97%) without purification. R_f = 0.78 (chloroform:ethanol 10:1); ^{1}H NMR (400 MHz, $CDCl_3$): δ = 2.61 (t, ^4J = 2.4 Hz, 1H, ≡CH), 4.00 (s, 3H, CH_3), 4.73 (s, 2H, $ArCH_2$), 4.83 (d, ^4J = 2.4 Hz, 2H, OCH_2), 7.31 (d, ^4J = 2.4 Hz, 1H, H_{Ar}), 7.69 (d, ^4J = 2.4 Hz, 1H, H_{Ar}) ppm; ^{13}C NMR (101 MHz, $CDCl_3$): δ = 46.4 ($ArCH_2$), 53.4 (CH_3), 56.3 (OCH_2), 76.6 (≡C), 77.5 (≡CH), 111.7 (CH_{Ar}), 112.8 (CH_{Ar}), 149.3 (C_{Ar}), 159.0 (C_{Ar}), 165.4 (C_{Ar}), 165.6 (C=O) ppm.

1.4. Dimethyl 4-(prop-2-yn-1-yloxy)pyridine-2,6-dicarboxylate ***SI-I***

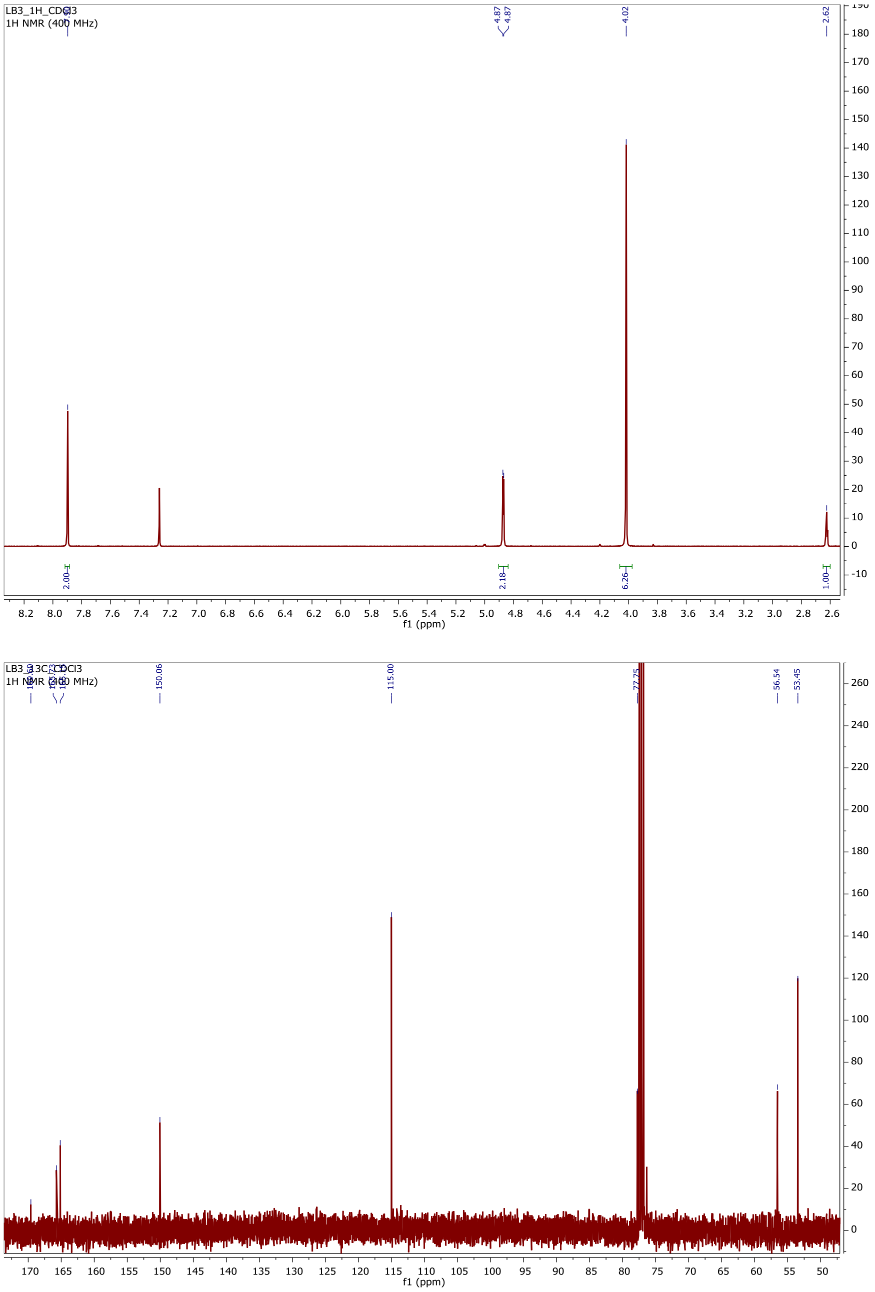

Figure S1. ^{1}H NMR and ^{13}C NMR spectra of compound **SI-I**.

1.4. Methyl 6-(hydroxymethyl)-4-(prop-2-yn-1-yloxy)picolinate ***SI-II***

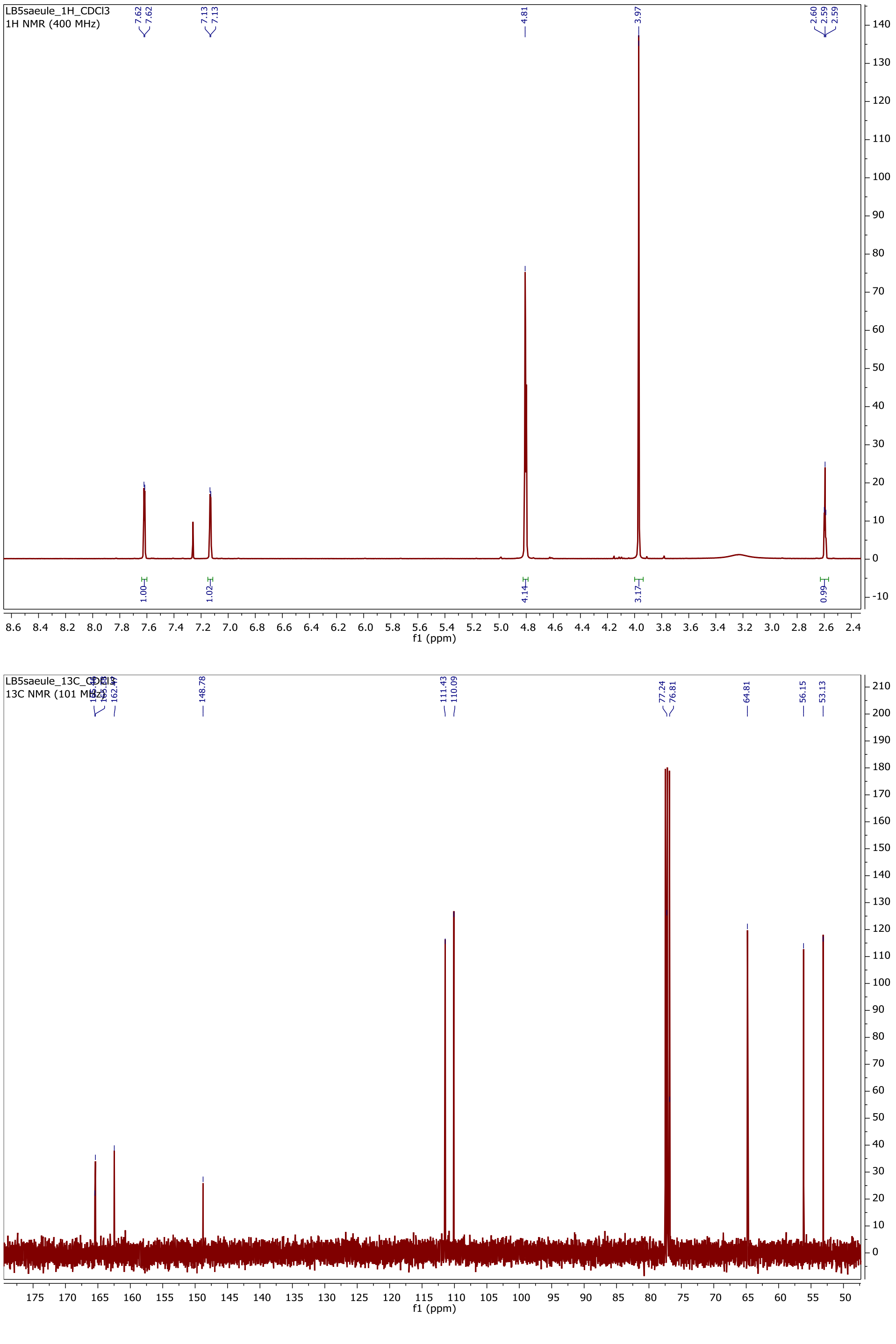

Figure S2. ^{1}H NMR and ^{13}C NMR spectra of compound **SI-II**.

1.5. Methyl 6-(chloromethyl)-4-(prop-2-yn-1-yloxy)picolinate ***SI-III***

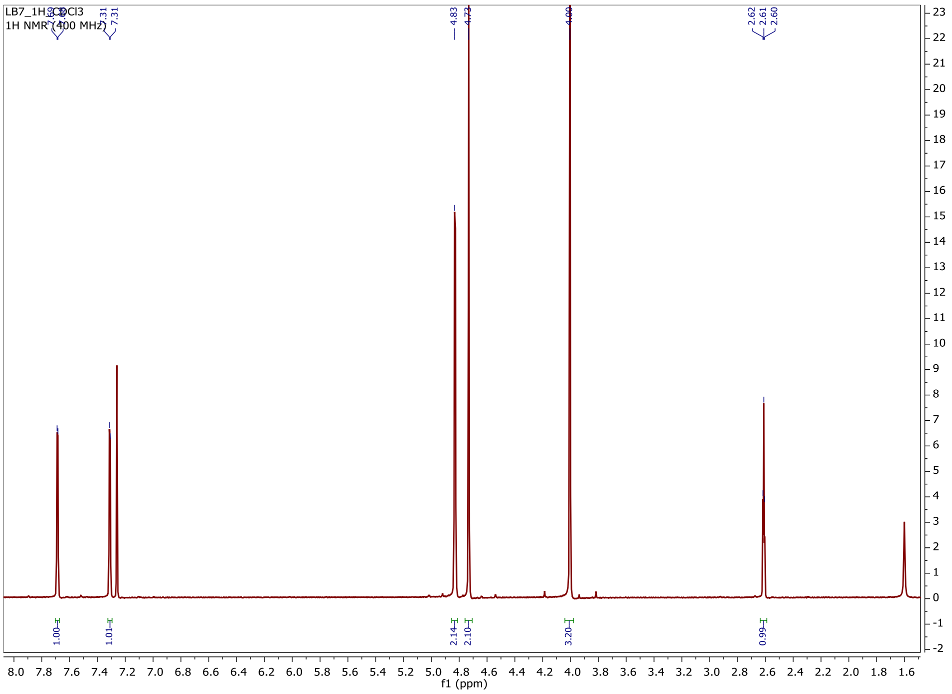

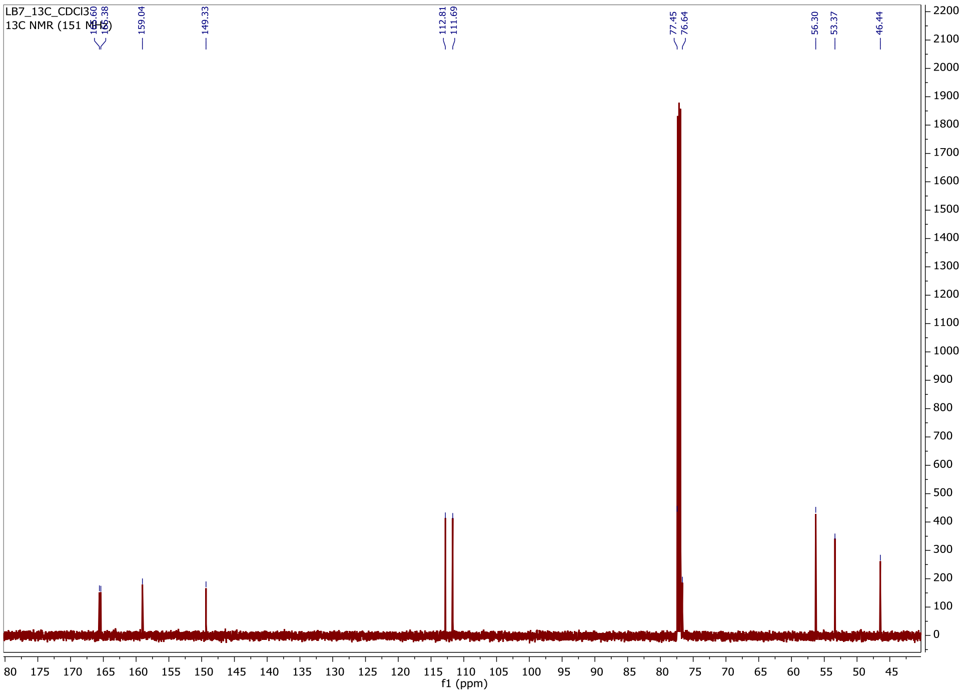

Figure S3. ^{1}H NMR and ^{13}C NMR spectra of compound **SI-III**.

2. Synthesis of the Model Chelator mcp-M-COOH

4-((1-(4-carboxybutyl)-1H-1,2,3-triazol-4-yl)methoxy)-6-((16-((6-carboxypyridin-2-yl)methyl)-1,4,10,13-tetraoxa-7,16-diazacyclooctadecan-7-yl)methyl)picolinic acid ***mcp-M-COOH***

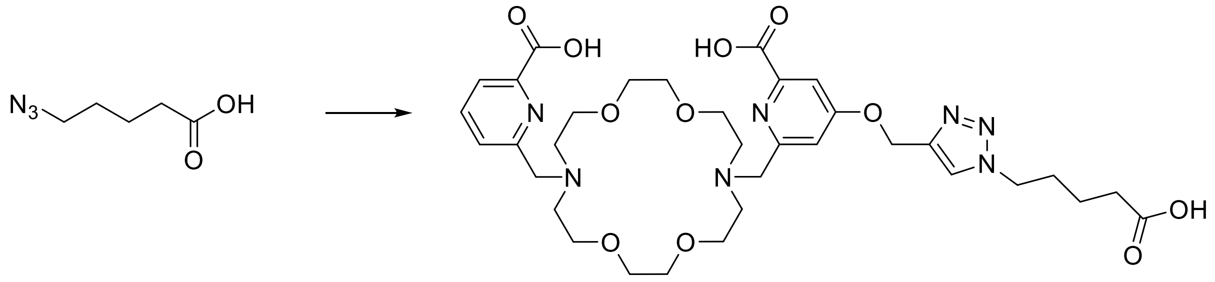

Scheme S2. Synthesis of the model chelator **mcp-M-COOH** under click chemistry conditions.

5-Azidobutyric acid (3.5 mg, 0.0244 mmol), **mcp-M-click** (20 mg, 0.0246 mmol), sodium ascorbate (49 mg, 0.0246 mmol) and TBTA-Cu(II)-complex (50 µL, 0.2 M, mixture from TBTA and $CuSO_4$) as catalyst were dissolved in water (3 mL) and stirred for 1.5 h. Afterwards, Na_2S was added to precipitate the Cu^{2+}. After filtration, the solvent was removed and the crude was purified via semin-preparative HPLC (6 mL/min, 10% → 35% ACN + 0.1% TFA in 35 min t_R = 21 min, Agilent Zorbax 300 SB-C18, 9.4 mm x 25 cm) to give **mcp-M-COOH** (4.3 mg, 83%).

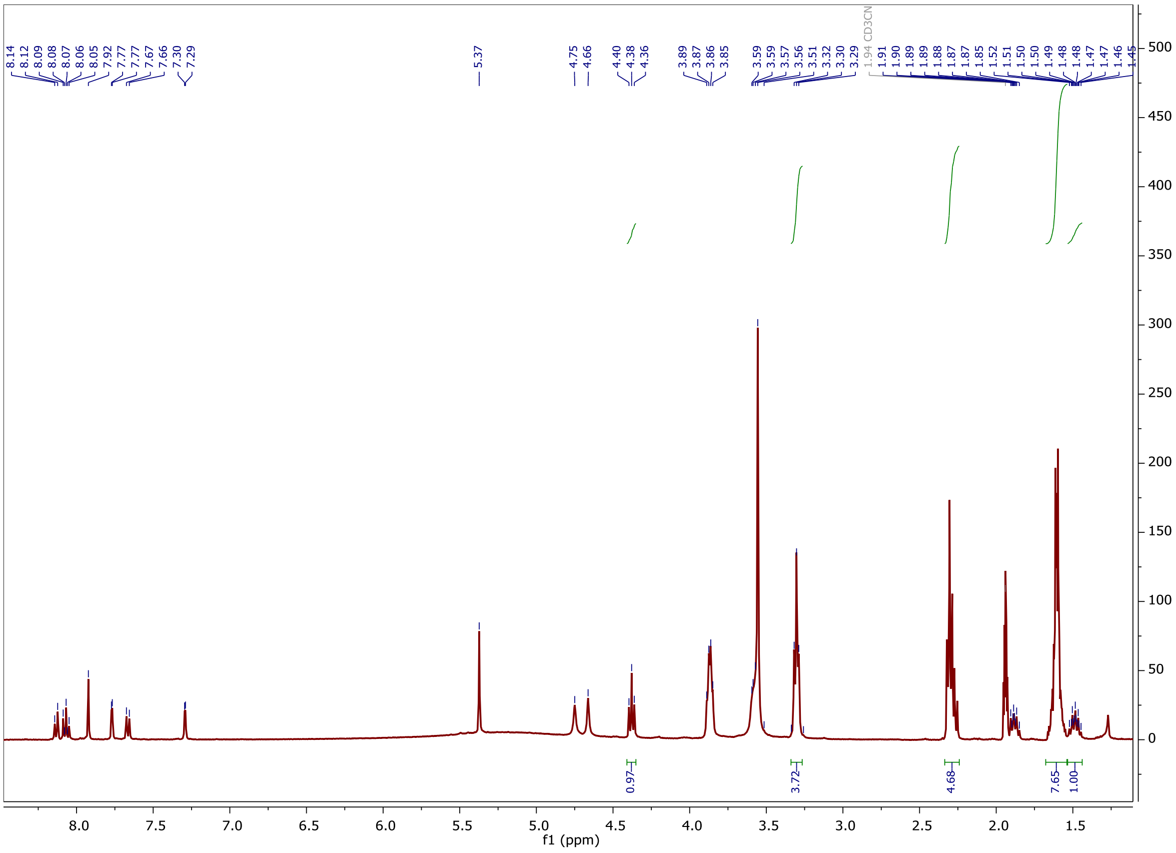

Figure S4. ^{1}H NMR spectrum of compound **mcp-M-COOH**.

3. Synthesis of the Azide-Functionalized PSMA-Derivative PSMA-N_3

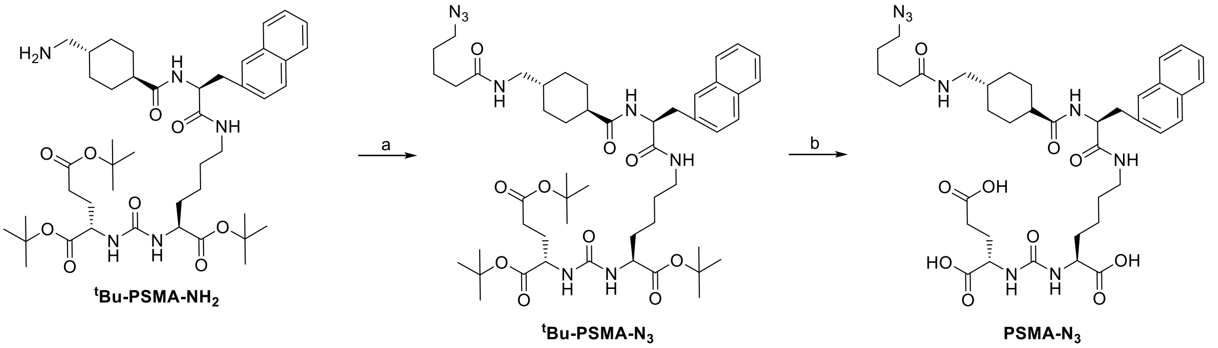

Scheme S3. Two-step synthesis approach for the click derivative **PSMA-N_3**.

3.1. tri-tert-butyl (3RS,10RS,14S)-1-((1r,4R)-4-((5-azidopentanamido)methyl)cyclohexyl)-3-(naphthalen-1-ylmethyl)-1,4,12-Trioxo-2,5,11,13-tetraazahexadecane-10,14,16-tricarboxylate ᵗBu-PSMA-N_3

EDC·HCl (43 mg, 0.23 mmol), 6-chloro-1-hydroxybenzotriazole (38 mg, 0,23 mmol), 5-azido-pentanoic acid (32 mg, 0.23 mmol) and DIPEA (29 mg, 0.23 mmol) were dissolved in anhydrous DMF (5 mL) at 0°C. **ᵗBu-PSMA-NH_2** (157 mg, 0.19 mmol) was dissolved in anhydrous DMF (5 mL) and slowly added to the mixture. The reaction was stirred at room temperature overnight. After reaction control via TLC, the solvent was removed and the crude product was purified by automated flash column chromatography (CH_2Cl_2/MeOH 100/0 → 98/2) to yield **ᵗBu-PSMA-N_3** as yellowish solid (150 mg, 83%). MS (ESI +): *m/z* = 949 [M^+]; 971 [M^++ Na].

*3.2.(3. RS,10RS,14S)-1-((1s,4S)-4-((5-azidopentanamido)methyl)cyclohexyl)-3-(naphthalen-1-ylmethyl)-1,4,12-Trioxo-2,5,11,13-tetraazahexadecane-10,14,16-tricarboxylic acid **PSMA-N_3***

ᵗBu-PSMA-N_3 (10 mg, 11 µmol) was dissolved in dichloromethane (1 mL). Trifluoro-acetic acid (3 mL) was added and the reaction was stirred overnight. The solvents were removed in an argon stream and the product was precipitated with ice-cold diethyl ether. The precipitate was washed with ice-cold n-pentane and ice-cold chloroform to yield **PSMA-N_3** as yellowish solid (9.5 mg, 100%). MS (ESI +): *m/z* = 782 [M^+ +H].

3.3. tri-tert-butyl (3RS,10RS,14S)-1-((1r,4R)-4-((5-azidopentanamido)methyl)cyclohexyl)-3-(naphthalen-1-ylmethyl)-1,4,12-Trioxo-2,5,11,13-tetraazahexadecane-10,14,16-tricarboxylate ***tBu-PSMA-N₃***

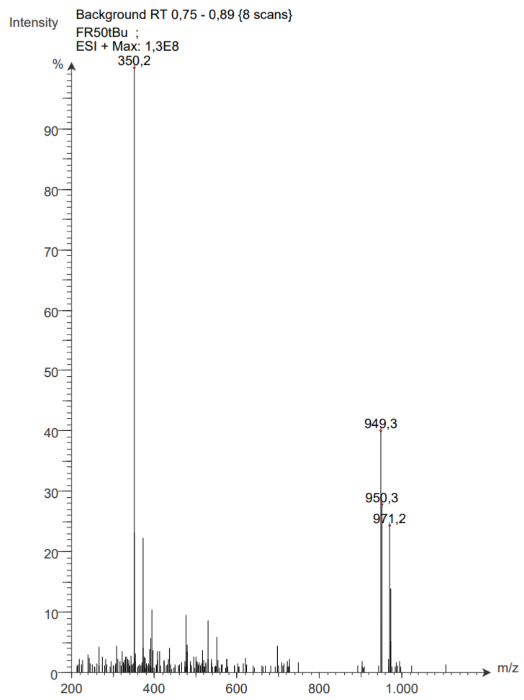

Figure S5. MS (ESI+) spectrum of compound **^{t}Bu-PSMA-N$_3$**.

3.4.(3. RS,10RS,14S)-1-((1s,4S)-4-((5-azidopentanamido)methyl)cyclohexyl)-3-(naphthalen-1-ylmethyl)-1,4,12-Trioxo-2,5,11,13-tetraazahexadecane-10,14,16-tricarboxylic Acid ***PSMA-N₃***

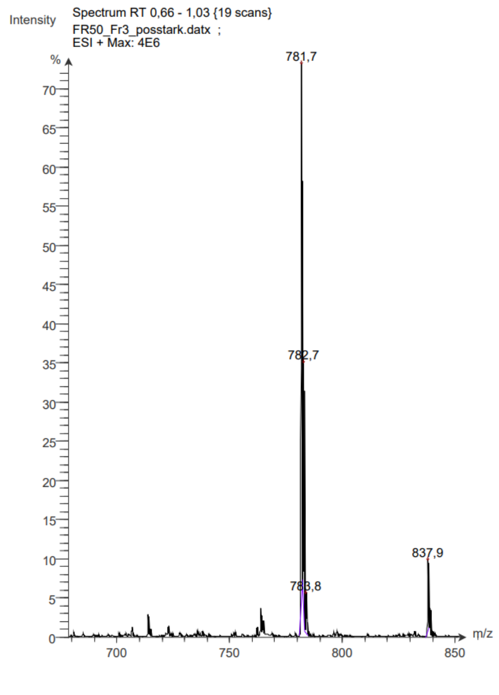

Figure S6. MS (ESI+) spectrum of compound **PSMA-N$_3$**.

4. NMR Spectra of Compounds 1 – 3, mcp-M-Click and mcp-D-Click

4.1. Methyl 6-((1,4,10,13-tetraoxa-7,16-diazacyclooctadecan-7-yl)methyl)picolinate **1**

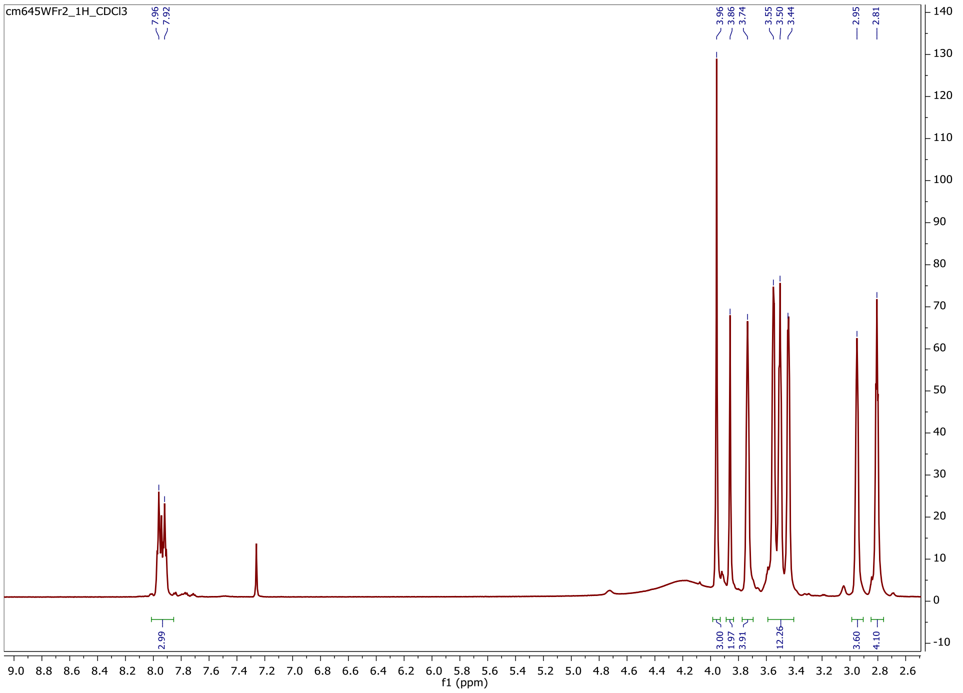

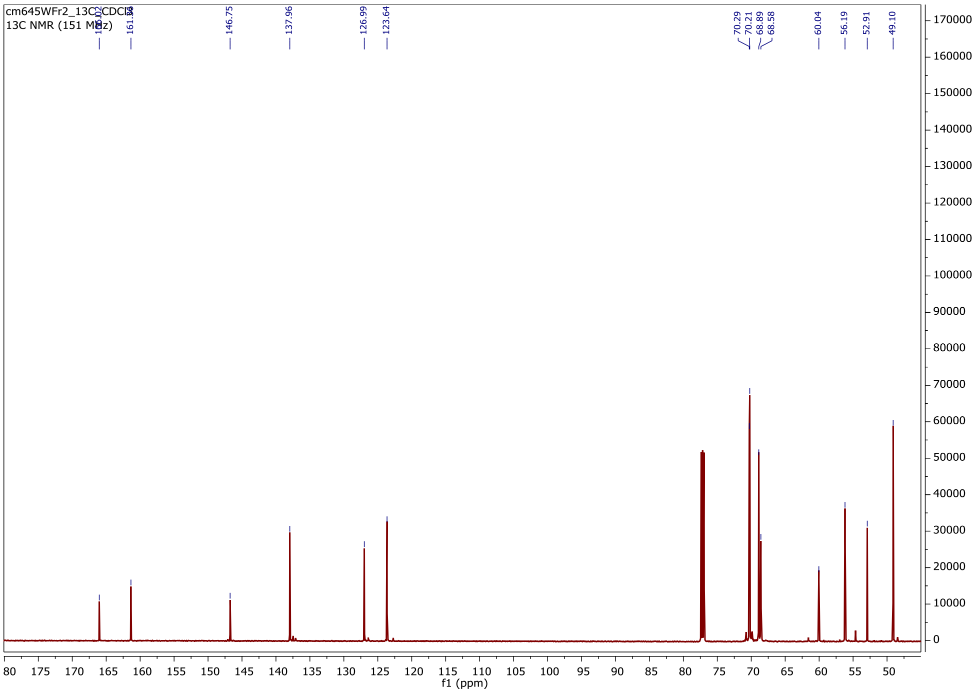

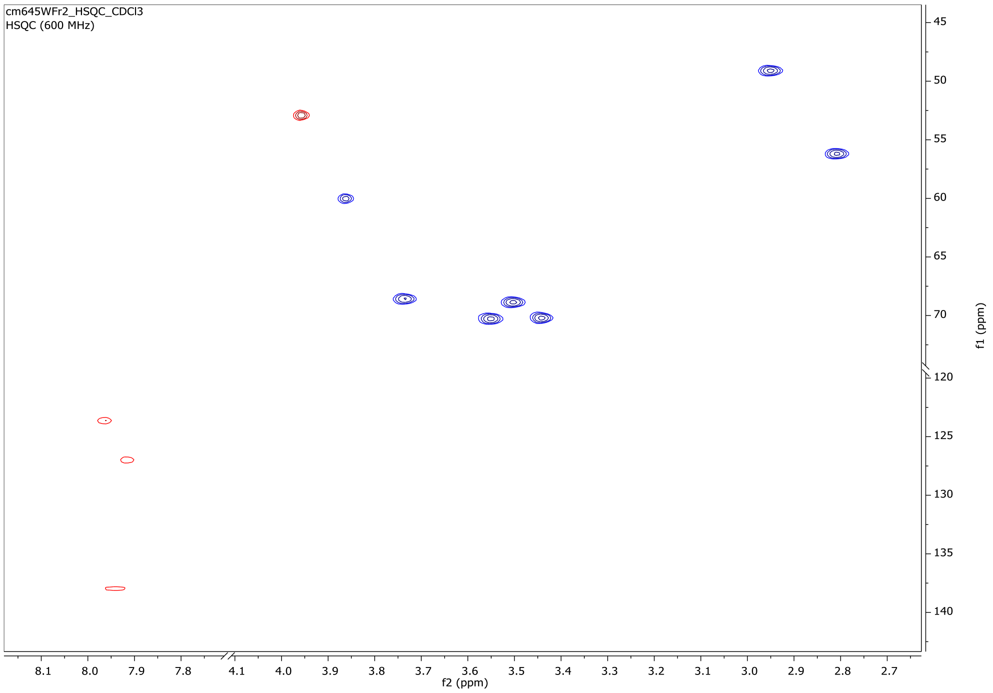

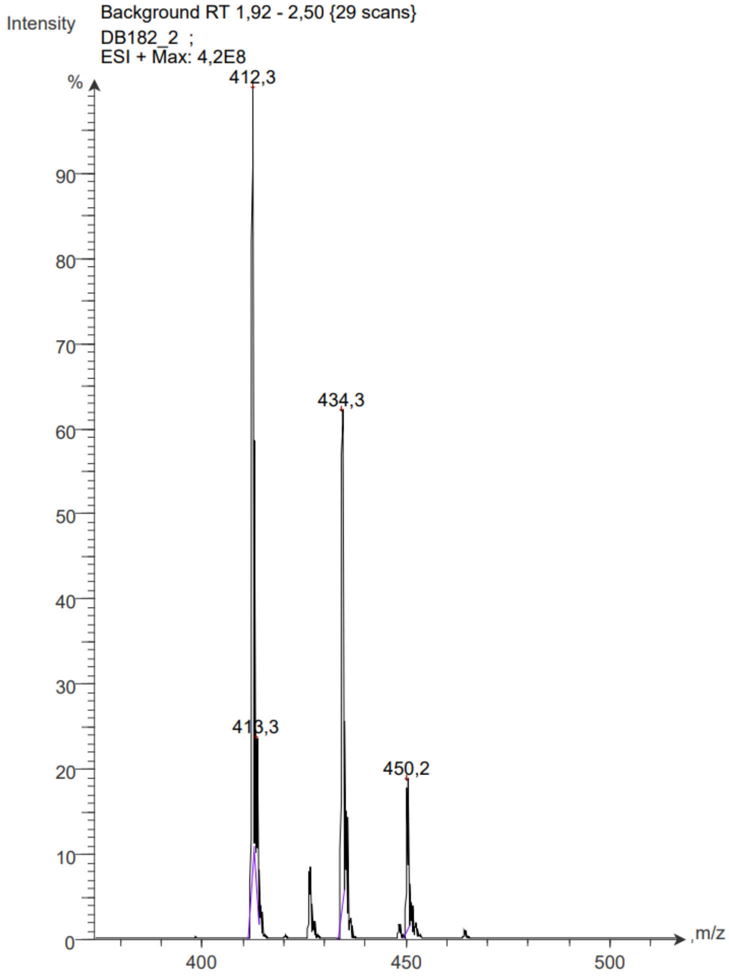

Figure S7. ^{1}H NMR, ^{13}C NMR, COSY, HSQC, and MS (ESI+) spectra of compound **1**.

4.2. Methyl 6-((16-((6-(methoxycarbonyl)pyridin-2-yl)methyl)-1,4,10,13-tetraoxa-7,16-diazacyclooctadecan-7-yl)methyl)-4-(prop-2-yn-1-yloxy)picolinate **2**

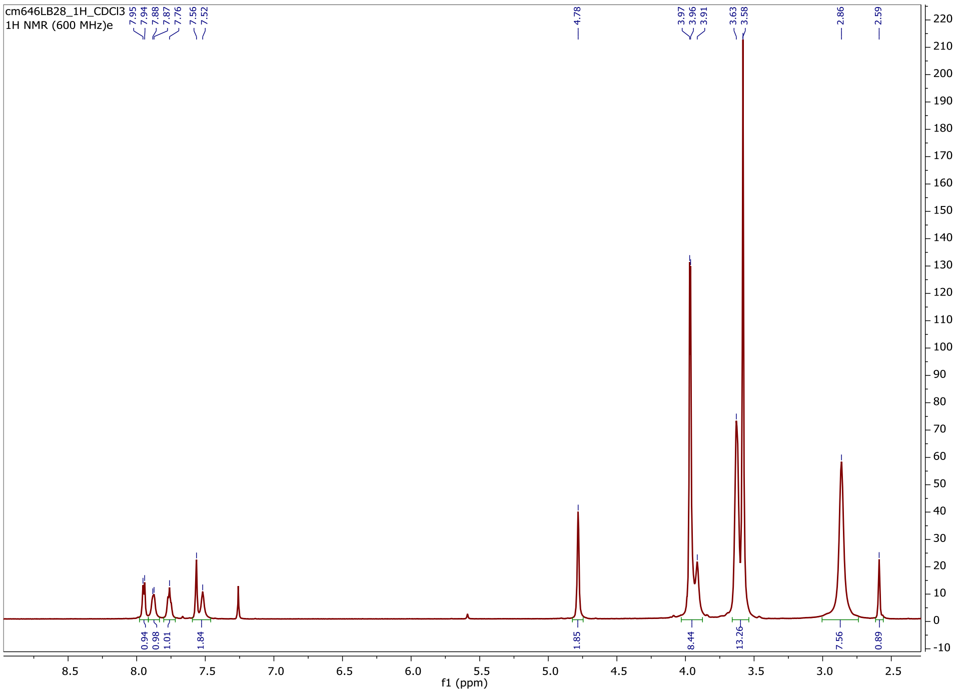

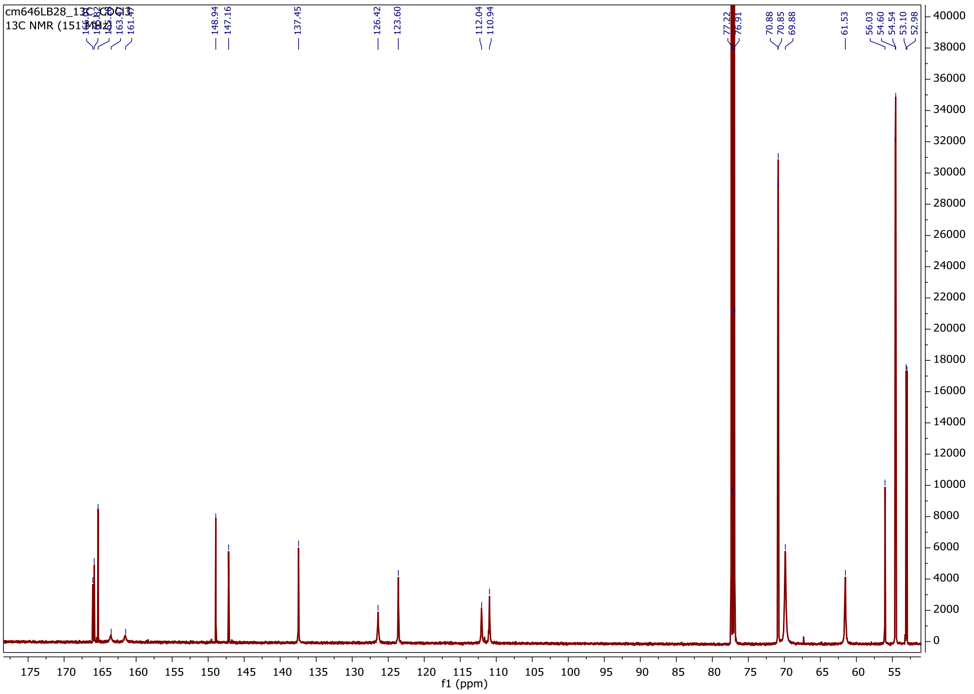

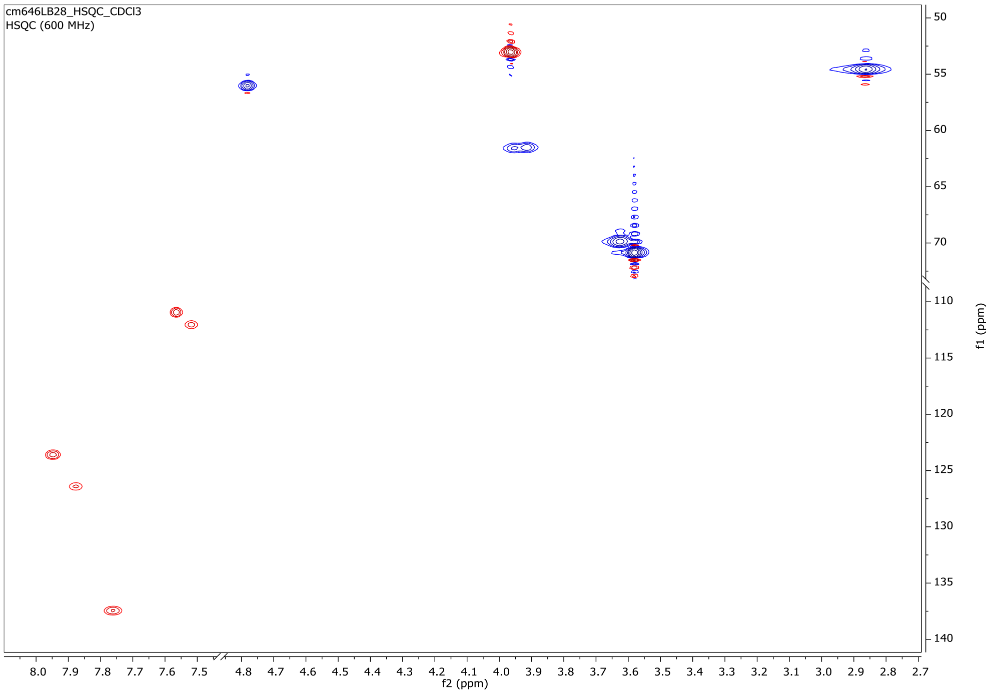

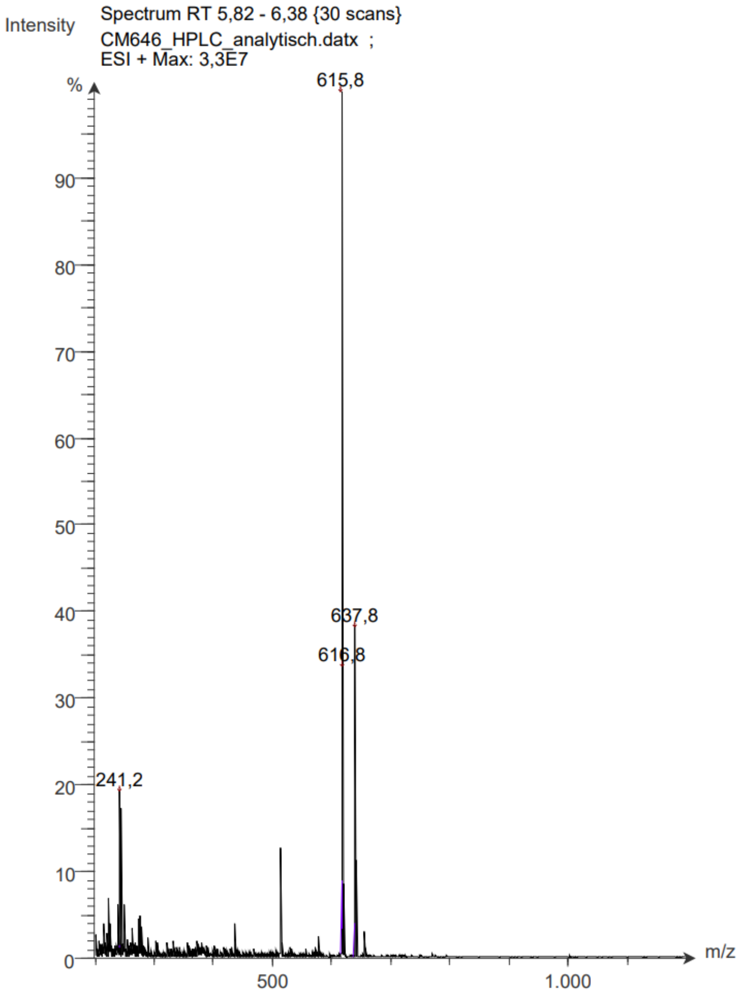

Figure S8. ^{1}H NMR, ^{13}C NMR, COSY, HSQC, and MS (ESI+) spectra of compound **2**.

4.3. Dimethyl 6,6′-((1,4,10,13-tetraoxa-7,16-diazacyclooctadecane-7,16-diyl)bis(methylene))bis(4-(prop-2-yn-1-yloxy)picolinate) **3**

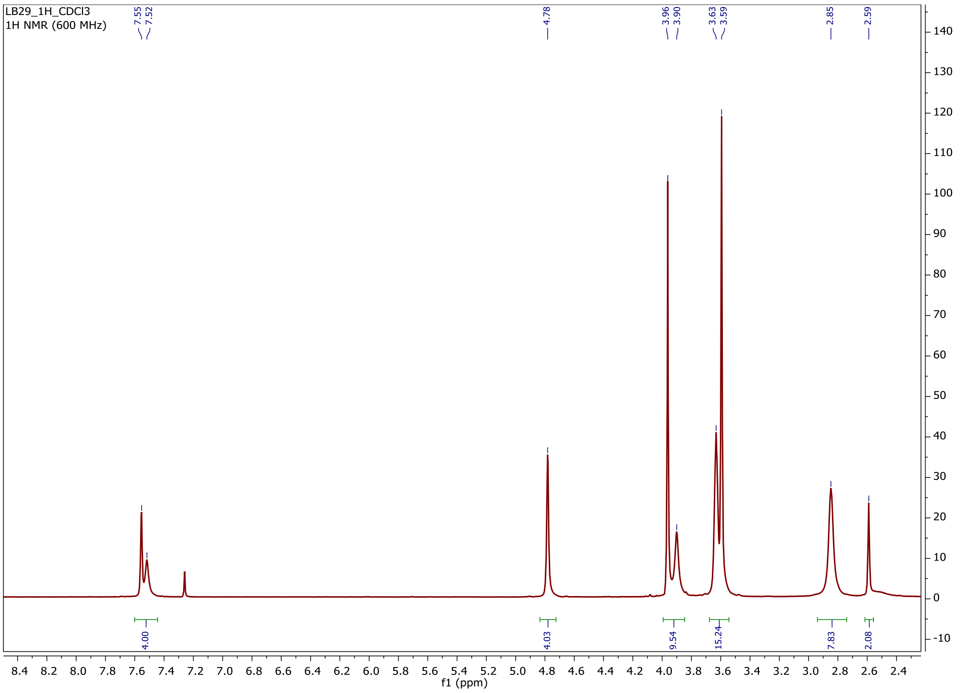

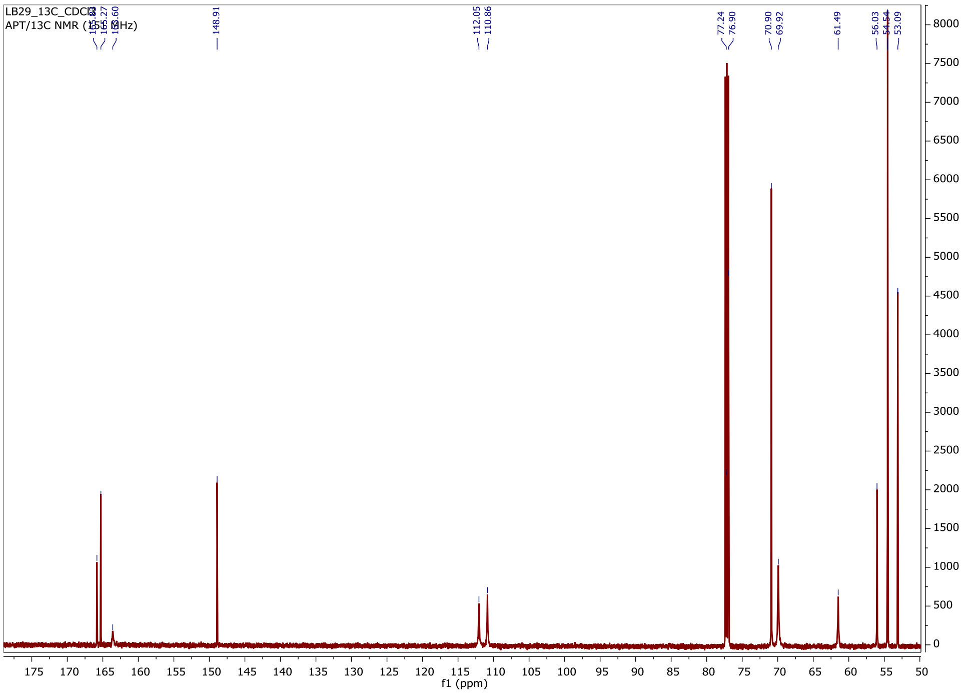

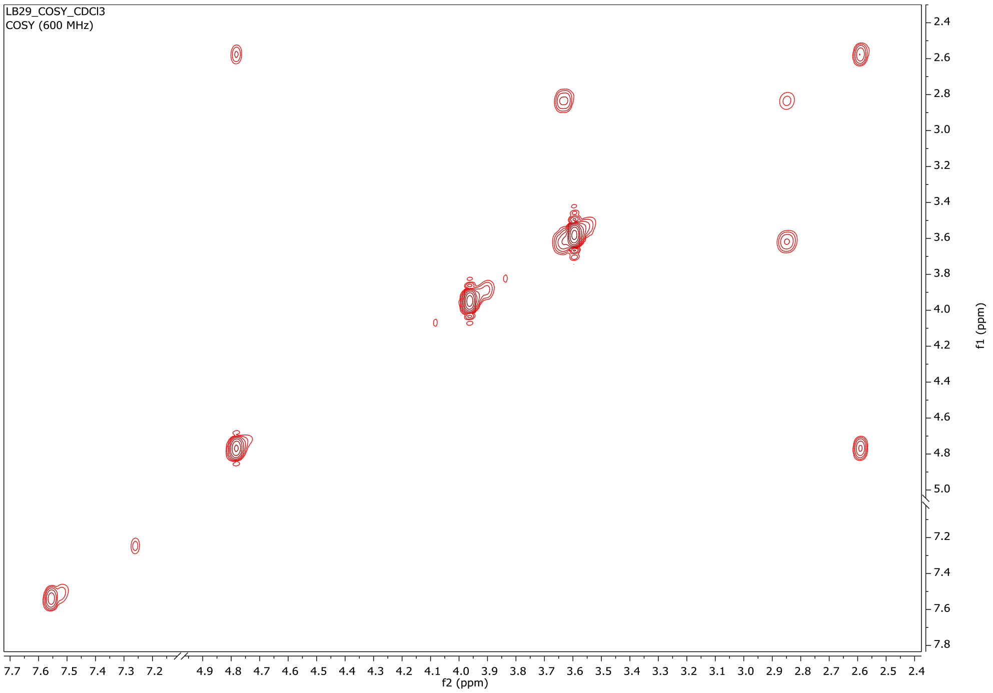
LB29_COSY_CDCl3
COSY (600 MHz)
f2 (ppm)
f1 (ppm)

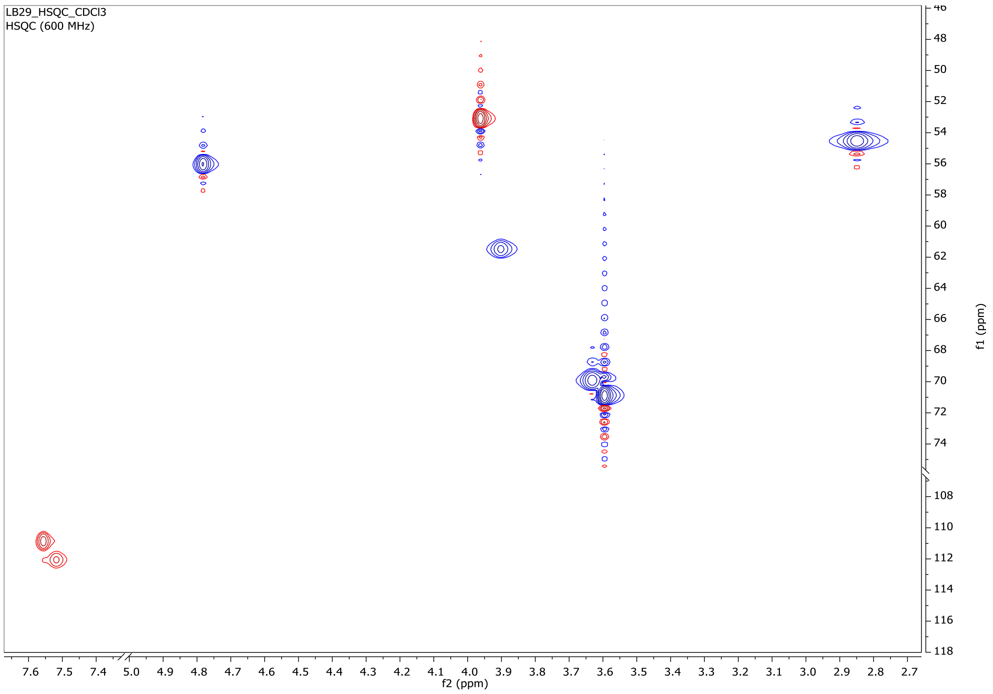
LB29_HSQC_CDCl3
HSQC (600 MHz)
f2 (ppm)
f1 (ppm)

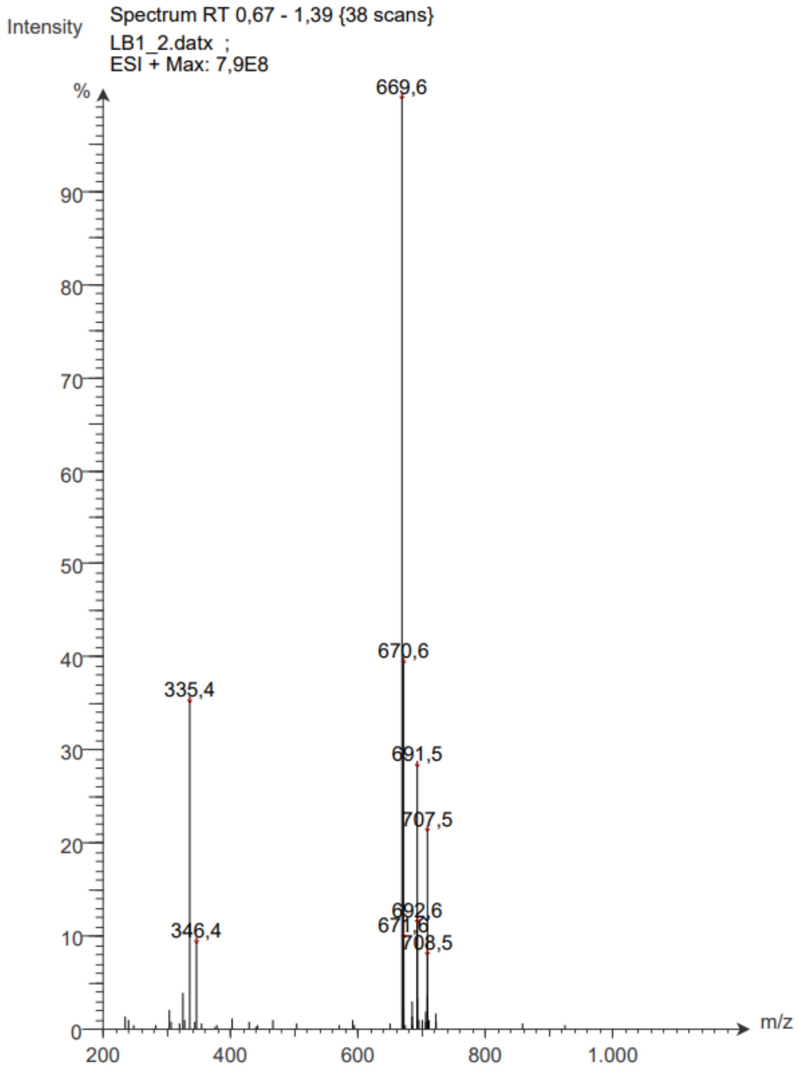

Figure S9. ^{1}H NMR, ^{13}C NMR, COSY, HSQC, and MS (ESI+) spectra of compound **3**.

4.4. mcp-M-Click

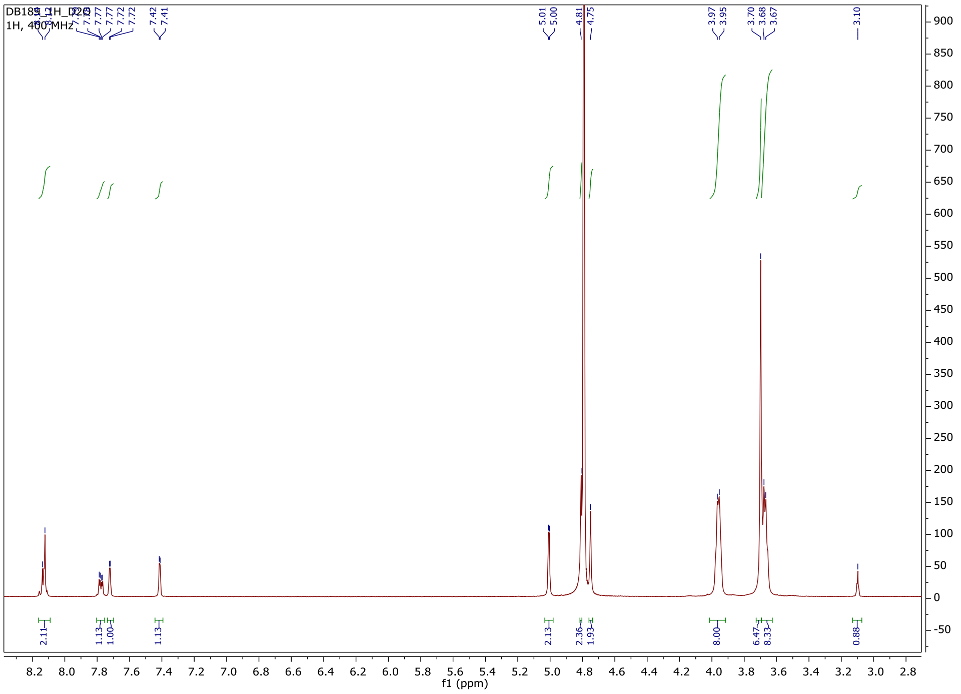

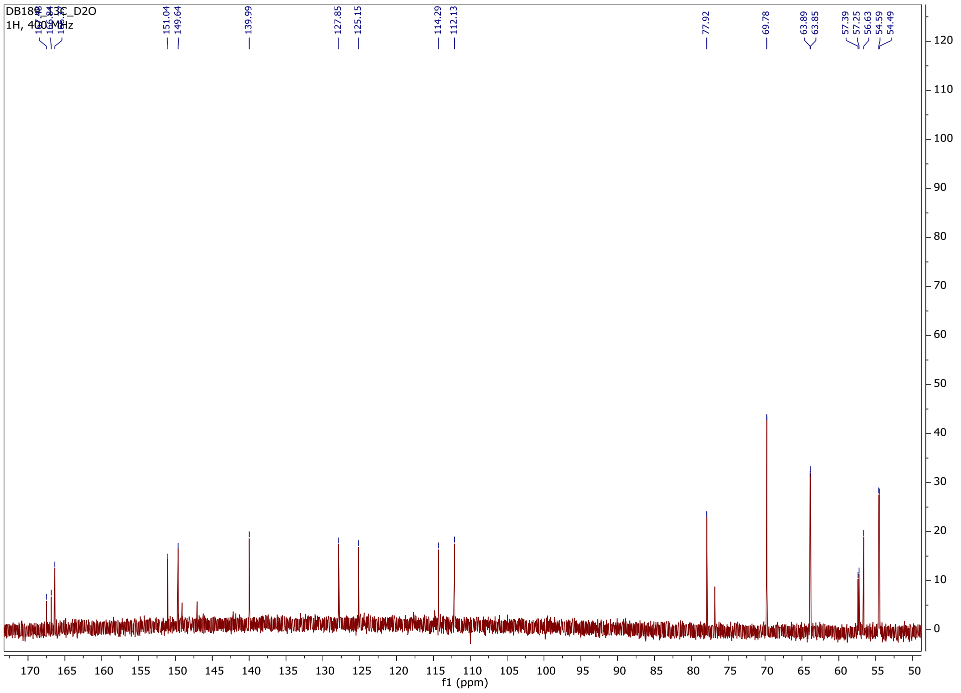

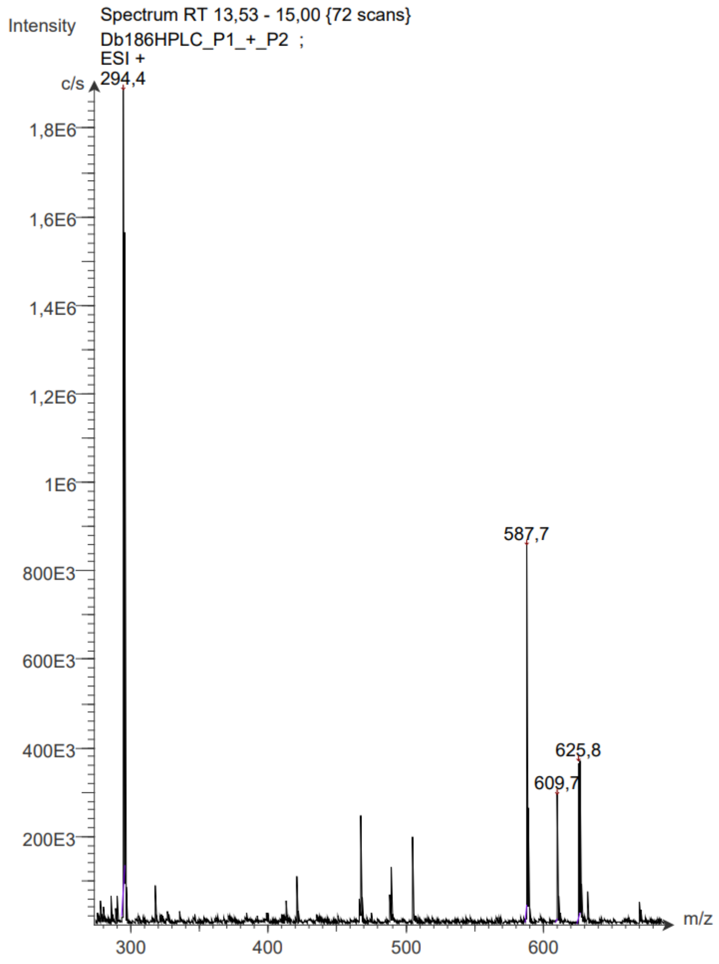

Figure S10. ^{1}H NMR, ^{13}C NMR, and MS (ESI+) spectra of compound **mcp-M-click**

4.5. mcp-D-Click

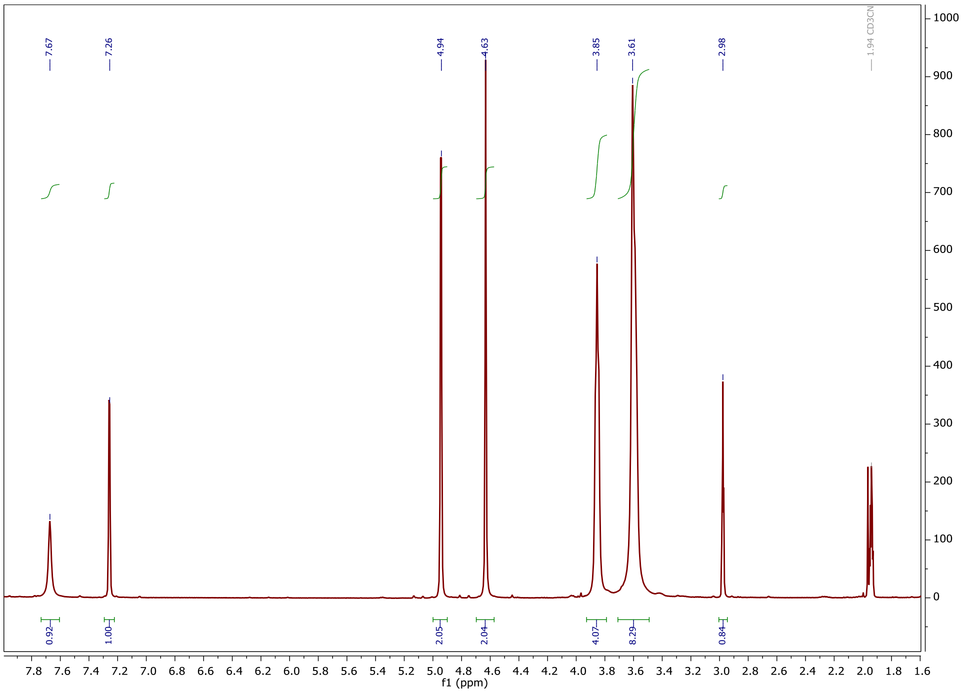

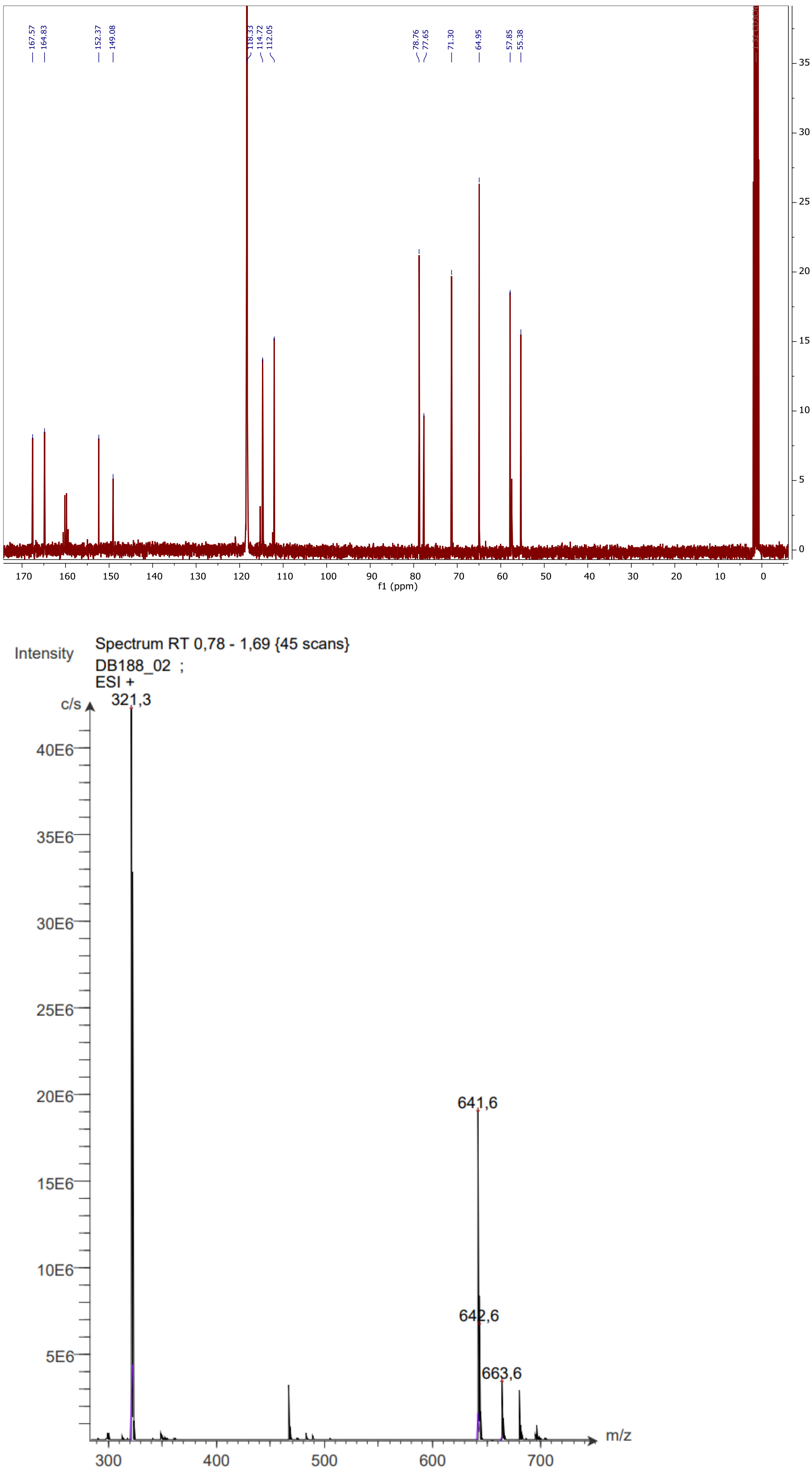

Figure S11. ^{1}H NMR, ^{13}C NMR, and MS (ESI+) spectra of compound **mcp-D-click**.

5. Mass Spectra of compounds mcp-M-PSMA and mcp-D-PSMA

5.1. mcp-M-PSMA

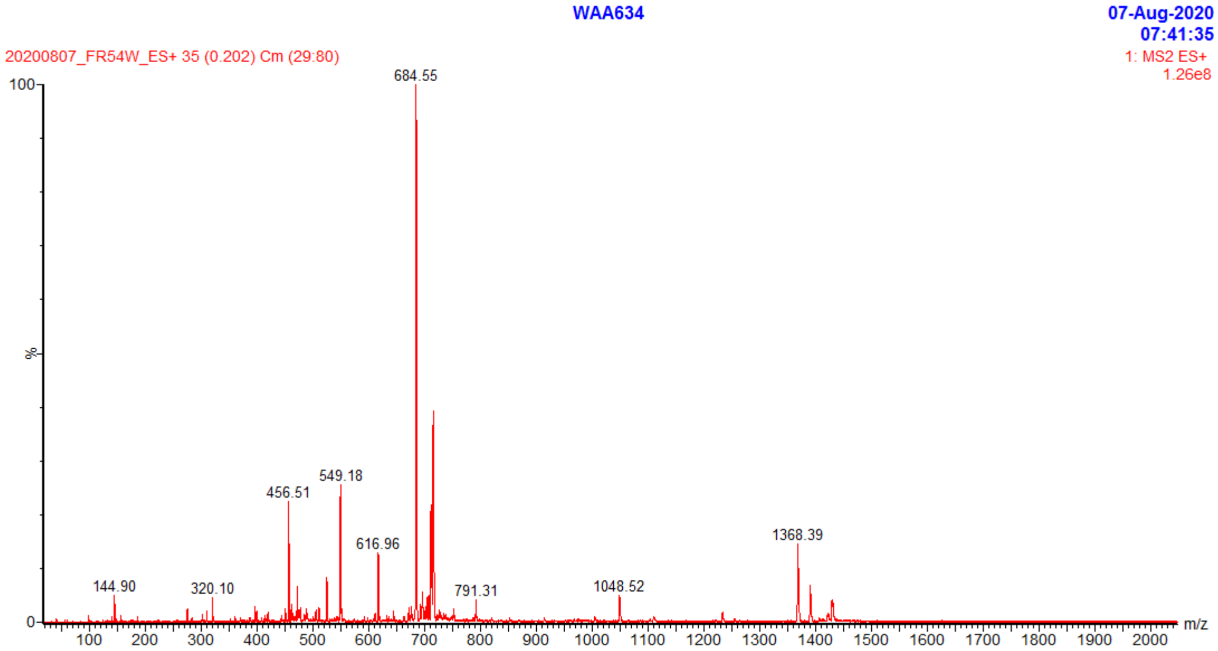

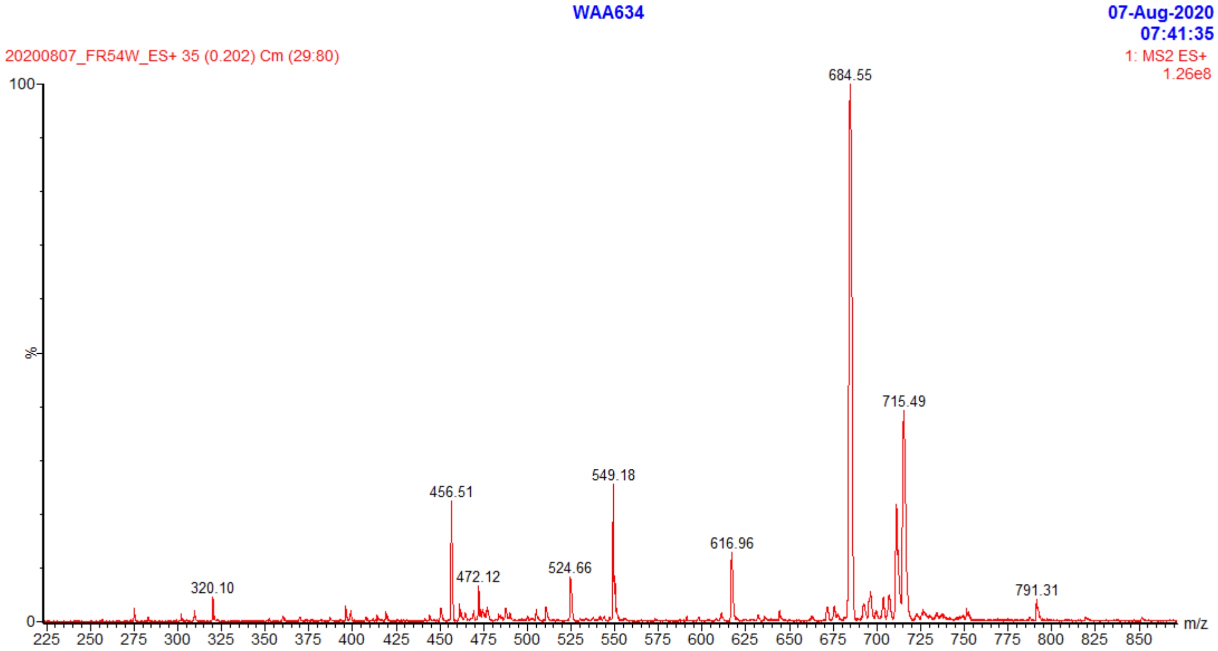

Figure S12. Mass spectrum (ESI+) and expanded region of ligand **mcp-M-PSMA**.

5.2. mcp-D-PSMA

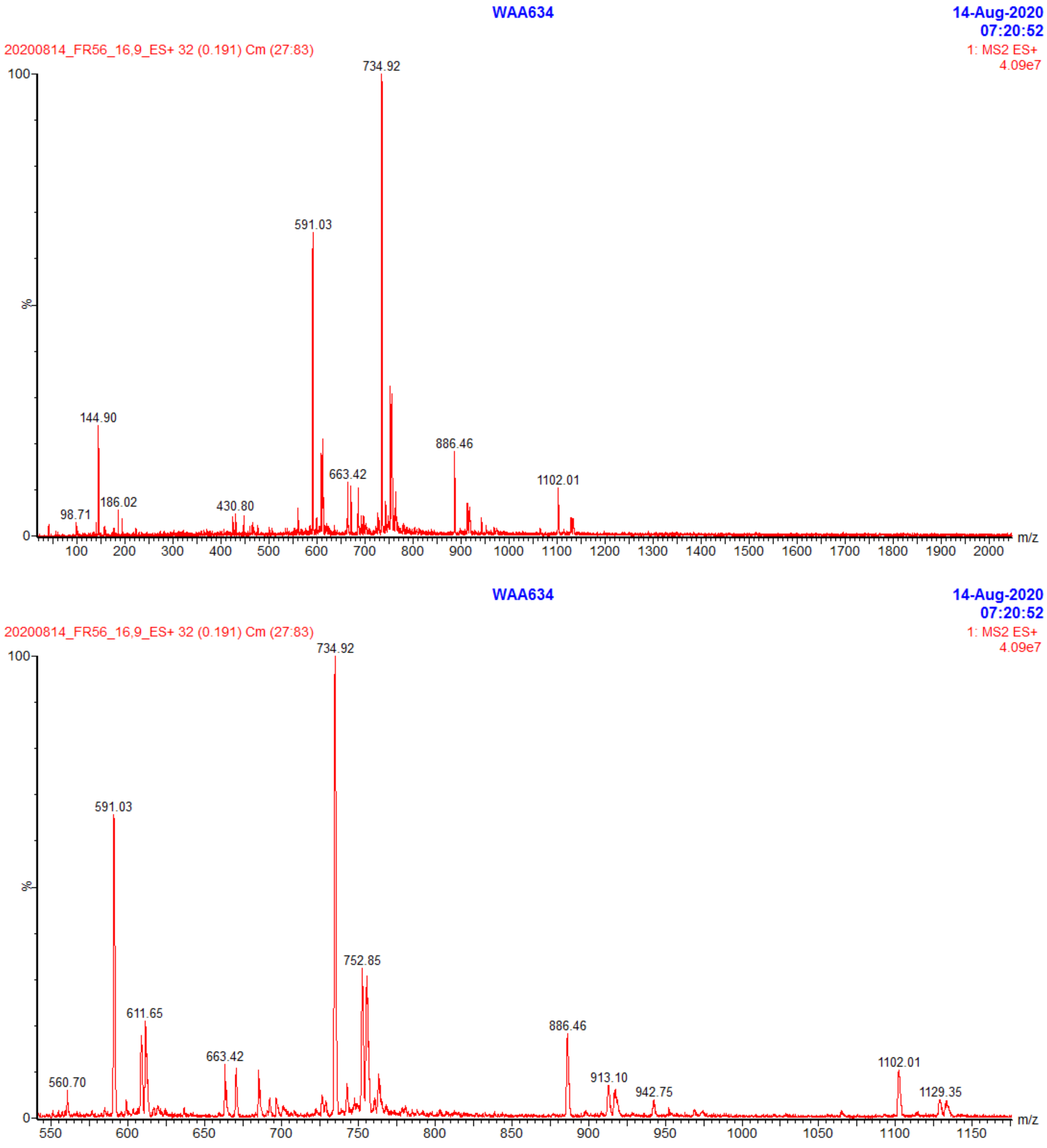

Figure S13. Mass spectrum (ESI+) and expanded region of ligand **mcp-D-PSMA**

6. Analysis of the Prostate-Specific Membrane Antigen (PSMA) Expression in Human Prostate Adenocarcinoma (LNCaP) Cells

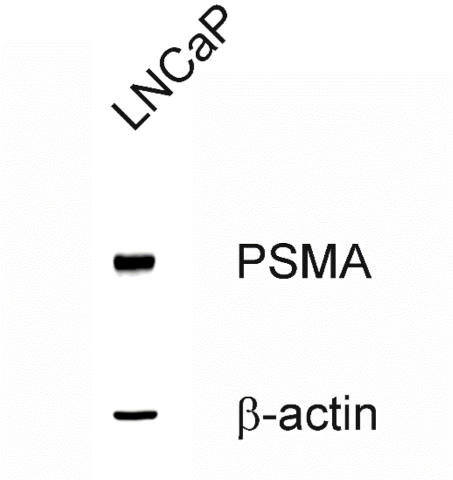

Figure S14. Analysis of the prostate-specific membrane antigen (PSMA) expression in human prostate adenocarcinoma (LNCaP) cells. Protein extracts of exponentially growing cells were prepared and proteins were separated by SDS-PAGE on a 12% polyacrylamide gel. After Western Blot transfer onto PVDF membranes, PSMA and β-actin proteins were detected by incubation with the respective specific antibodies followed by secondary HRP-coupled antibodies and chemiluminescence detection. The corresponding uncropped blots are shown in Figure S16.

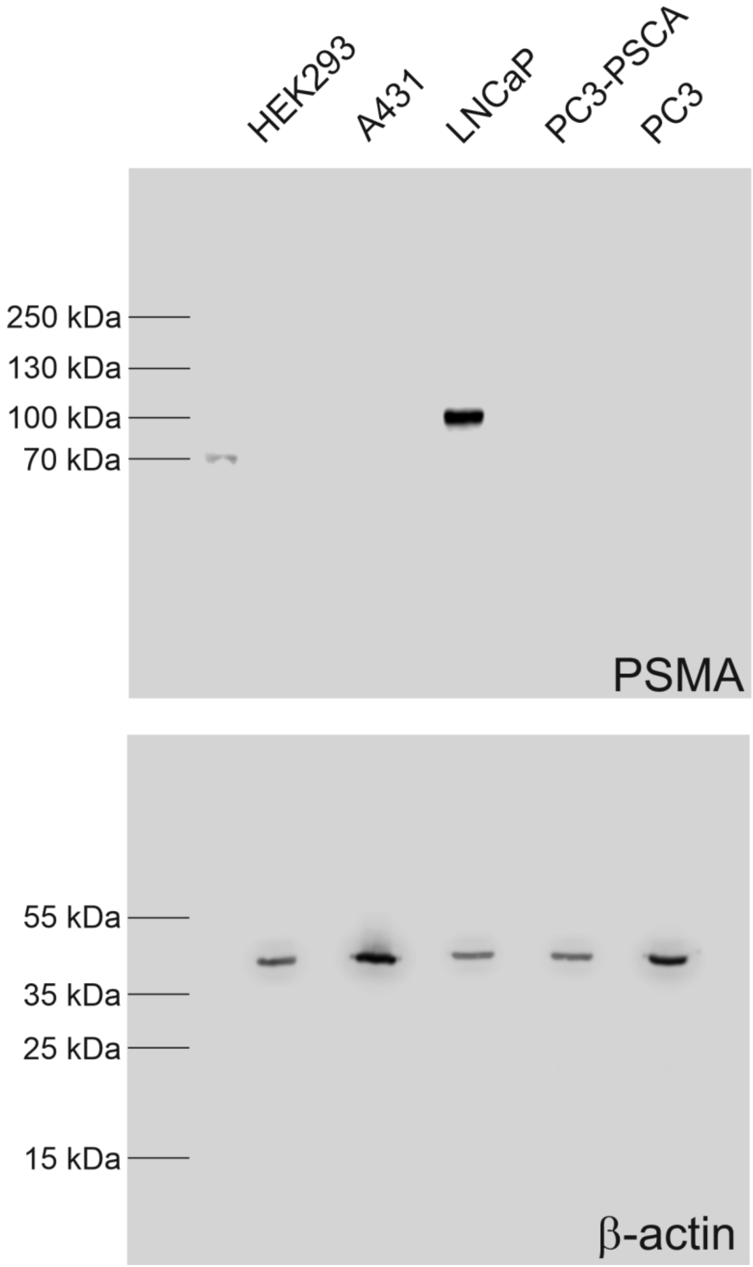

Figure S15. Analysis of the prostate-specific membrane antigen (PSMA) expression in HEK293, A431, LNCaP, PC3-PSCA, and PC3 cells. Protein extracts of exponentially growing cells were prepared and proteins were separated by SDS-PAGE on a 12% polyacrylamide gel. After Western Blot transfer onto PVDF membranes, PSMA and β-actin proteins were detected by incubation with the respective specific antibodies followed by secondary HRP-coupled antibodies and chemiluminescence detection.

7. Results of the Colony Forming Assay Showing the Survival of LNCaP Cells after Treatment with the ^{225}Ac-Radioconjugates.

Table S1. Percentage amount of colonies referred to the non-radioactive control.

Activity Concentration	[^{225}Ac]Ac-mcp-M-PSMA	[^{225}Ac]Ac-mcp-D-PSMA	[^{225}Ac]Ac^{3+}
1 h incubation time			
0.05 kBq/mL	107.2%	75.0%	114.7%
0.5 kBq/mL	91.8%	3.8%	90.5%
5 kBq/mL	19.4%	3.1%	32.9%
50 kBq/mL	1.4%	0.8%	2.5%
4 h incubation time			
0.05 kBq/mL	116.8%	49.1%	87.1%
0.5 kBq/mL	57.2%	6.2%	83.8%
5 kBq/mL	5.3%	0.5%	13.0%
50 kBq/mL	0.9%	0.4%	1.7%

8. Biodistribution Study of [^{225}Ac]Ac-mcp-M-PSMA and [^{225}Ac]Ac-mcp-D-PSMA in LNCaP Tumor-Bearing SCID Mice

*8.1. Biodistribution of [^{225}Ac]Ac-**mcp-M-PSMA** in LNCaP Tumor-Bearing SCID Mice*

Table S2. Quantitative evaluation of [^{225}Ac]Ac-**mcp-M-PSMA** in LNCaP tumor-bearing SCID mice. The values are given as the mean of the percentage of injected dose per gram of organ ± standard deviation (*n* = 4).

Organ/Tissue	10 min p.i.		1 h p.i.		24 h p.i.	
	%ID/g	*SD*	%ID/g	*SD*	%ID/g	*SD*
blood	6.85	0.58	0.73	0.16	0.04	0.00
spleen	25.05	10.42	2.90	1.69	0.37	0.24
pancreas	1.12	0.13	0.26	0.08	0.02	0.01
stomach	2.52	0.43	0.46	0.11	0.50	0.67
intestine	1.37	0.14	1.31	0.14	0.35	0.21
kidneys	117.81	19.86	67.84	23.92	1.35	0.26
liver	2.49	0.17	0.62	0.04	0.27	0.04
heart	2.54	0.40	0.36	0.07	0.08	0.03
lung	5.83	0.42	0.76	0.16	0.10	0.06
muscle	1.21	0.22	0.23	0.03	0.02	0.01
bone	1.18	0.51	1.80	1.22	0.18	0.12
tumor	10.01	2.86	12.52	2.73	6.78	0.45

*8.2. Biodistribution of [^{225}Ac]Ac-**mcp-D-PSMA** in LNCaP Tumor-Bearing SCID Mice*

Table S3. Quantitative evaluation of [^{225}Ac]Ac-**mcp-D-PSMA** in LNCaP tumor-bearing SCID mice. The values are given as the mean of the percentage of injected dose per gram of organ ± standard deviation (*n* = 4).

Organ/Tissue	10 min p.i.		1 h p.i.		24 h p.i.	
	%ID/g	*SD*	%ID/g	*SD*	%ID/g	*SD*
blood	7.52	0.65	2.72	0.24	0.08	0.02
spleen	13.42	6.11	4.57	1.71	0.41	0.28
pancreas	0.82	0.12	0.36	0.06	0.05	0.02
stomach	1.66	0.23	0.68	0.12	0.14	0.04
intestine	0.89	0.07	0.44	0.05	0.13	0.06
kidneys	66.01	7.20	103.03	24.68	7.17	1.96

liver	1.66	0.12	0.76	0.04	0.59	0.03
heart	2.61	0.18	1.00	0.20	0.12	0.01
lung	5.51	0.24	1.99	0.24	0.12	0.03
muscle	0.91	0.10	0.29	0.02	0.04	0.02
bone	0.94	0.08	0.64	0.14	0.33	0.13
tumor	4.48	0.83	8.72	1.77	12.21	4.31

9. Biodistribution Study of [^{225}Ac]Ac-mcp-M-PSMA and [^{225}Ac]Ac-mcp-D-PSMA in Healthy Mice

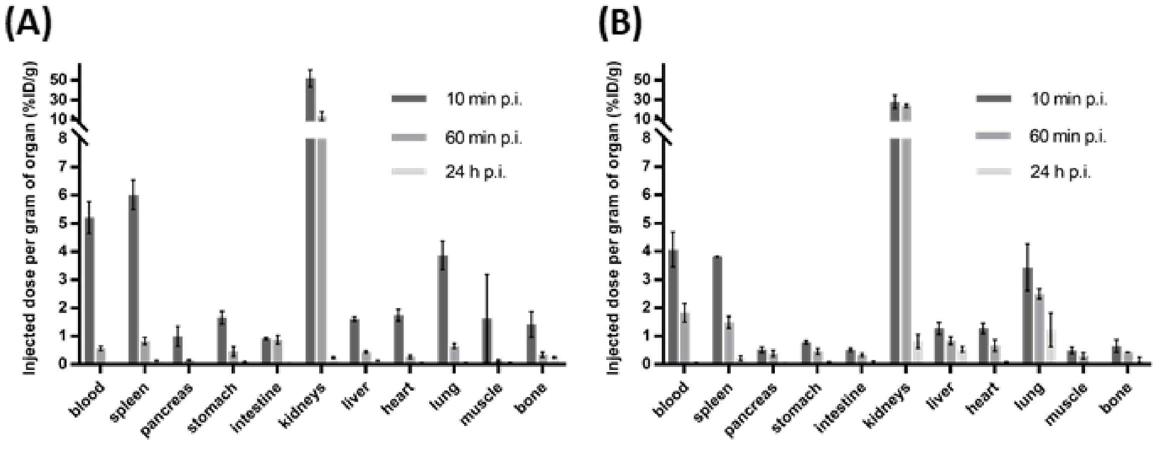

Figure S16. Biodistribution of [^{225}Ac]Ac-**mcp-M-PSMA** (**A**) and [^{225}Ac]Ac-**mcp-D-PSMA** (**B**) using healthy SCID mice 10 min. 60 min. and 24 h p.i. The data are presented as the mean of the percentage of injected dose per gram of organ ± standard deviation (n = 4).

*9.1. Biodistribution of [^{225}Ac]Ac-**mcp-M-PSMA** in Healthy Mice*

Table S4. Quantitative evaluation of [^{225}Ac]Ac-**mcp-M-PSMA** in healthy mice. The values are given as the mean of the percentage of injected dose per gram of organ ± standard deviation (n = 4).

**Organ/Tissue**	**10 min p.i.**		**1 h p.i.**		**24 h p.i.**	
	**%ID/g**	***SD***	**%ID/g**	***SD***	**%ID/g**	***SD***
blood	5.20	0.57	0.56	0.08	0.02	0.00
spleen	6.02	0.53	0.83	0.13	0.13	0.02
pancreas	0.99	0.36	0.14	0.03	0.02	0.01
stomach	1.66	0.22	0.45	0.18	0.07	0.03
intestine	0.91	0.04	0.87	0.15	0.03	0.00
kidneys	51.93	8.63	13.68	4.51	0.23	0.03
liver	1.61	0.08	0.44	0.05	0.13	0.01
heart	1.75	0.21	0.27	0.06	0.05	0.01
lung	3.86	0.51	0.65	0.09	0.04	0.01
muscle	1.63	1.56	0.11	0.04	0.05	0.02
bone	1.41	0.45	0.34	0.09	0.25	0.03

*9.2. Biodistribution of [^{225}Ac]Ac-**mcp-D-PSMA** in Healthy Mice*

Table S5. Quantitative evaluation of [^{225}Ac]Ac-**mcp-D-PSMA** in healthy mice. The values are given as the mean of the percentage of injected dose per gram of organ ± standard deviation (*n* = 4).

**Organ/Tissue**	**10 min p.i.**		**1 h p.i.**		**24 h p.i.**	
	**%ID/g**	***SD***	**%ID/g**	***SD***	**%ID/g**	***SD***
blood	4.07	0.62	1.83	0.32	0.05	0.01
spleen	3.82	0.01	1.49	0.21	0.22	0.09
pancreas	0.52	0.10	0.38	0.11	0.03	0.01
stomach	0.78	0.06	0.46	0.10	0.08	0.01
intestine	0.51	0.06	0.33	0.06	0.07	0.04
kidneys	27.99	6.83	23.95	1.80	0.82	0.24
liver	1.27	0.21	0.84	0.14	0.54	0.09
heart	1.28	0.18	0.67	0.20	0.07	0.02
lung	3.44	0.83	2.50	0.18	1.22	0.60
muscle	0.49	0.12	0.30	0.11	0.02	0.01
bone	0.65	0.22	0.43	0.00	0.15	0.10
